# Functional Domains and Evolutionary History of the PMEL and GPNMB Family Proteins

**DOI:** 10.3390/molecules26123529

**Published:** 2021-06-09

**Authors:** Paul W. Chrystal, Tim Footz, Elizabeth D. Hodges, Justin A. Jensen, Michael A. Walter, W. Ted Allison

**Affiliations:** 1 Department of Medical Genetics, University of Alberta, Edmonton, AB T6G 2R3, Canada; pchrysta@ualberta.ca (P.W.C.); tfootz@ualberta.ca (T.F.); 2 Department of Biological Sciences, University of Alberta, Edmonton, AB T7Y 1C4, Canada; edhodges@ualberta.ca (E.D.H.); jajensen@ualberta.ca (J.A.J.); 3 Centre for Prions & Protein Folding Disease, University of Alberta, Edmonton, AB T6G 2M8, Canada

**Keywords:** PKAT family proteins, PMEL-17-related family PTHR11861, functional amyloid, melanosomes, pigmentation, neurodegeneration

## Abstract

The ancient paralogs premelanosome protein (*PMEL*) and glycoprotein nonmetastatic melanoma protein B (*GPNMB*) have independently emerged as intriguing disease loci in recent years. Both proteins possess common functional domains and variants that cause a shared spectrum of overlapping phenotypes and disease associations: melanin-based pigmentation, cancer, neurodegenerative disease and glaucoma. Surprisingly, these proteins have yet to be shown to physically or genetically interact within the same cellular pathway. This juxtaposition inspired us to compare and contrast this family across a breadth of species to better understand the divergent evolutionary trajectories of two related, but distinct, genes. In this study, we investigated the evolutionary history of *PMEL* and *GPNMB* in clade-representative species and identified *TMEM130* as the most ancient paralog of the family. By curating the functional domains in each paralog, we identified many commonalities dating back to the emergence of the gene family in basal metazoans. *PMEL* and *GPNMB* have gained functional domains since their divergence from *TMEM130*, including the core amyloid fragment (CAF) that is critical for the amyloid potential of PMEL. Additionally, the *PMEL* gene has acquired the enigmatic repeat domain (RPT), composed of a variable number of imperfect tandem repeats; this domain acts in an accessory role to control amyloid formation. Our analyses revealed the vast variability in sequence, length and repeat number in homologous RPT domains between craniates, even within the same taxonomic class. We hope that these analyses inspire further investigation into a gene family that is remarkable from the evolutionary, pathological and cell biology perspectives.

## 1. Introduction

In recent years, the disease-causing genes *GPNMB* (Glycoprotein nonmetastatic melanoma protein B) (i.e., HGFIN, NMB and Osteoactivin) and *PMEL* (premelanosome protein) (i.e., PMEL17, SILV, MMP115 and gp100) have generated increased interest from disparate biomedical fields. *GPNMB* and *PMEL* are related genes that encode for proteins with a common functional domain architecture: a signal peptide, core amyloid fragment, Kringle-like domain and a single transmembrane domain [1]. Consistent with this common architecture, both have also been implicated in the same diverse biological roles via their association with phenotypic presentation and disease, i.e., cancer [2,3,4,5,6], glaucoma [7,8,9], neurodegenerative disease [10,11,12,13], melanin-based pigmentation [14,15,16,17] and amyloid formation/amyloidosis [18,19,20,21,22]. It is therefore surprising that these related proteins with a strikingly similar suite of functional domains have yet to be identified as interacting in, or being partially redundant in, any biological process or pathway.

PMEL is a single-pass Type I transmembrane glycoprotein expressed in pigmented melanocytes, retinal pigmented epithelium and the substantia nigra [5]. This protein is processed through several complex steps of O-linked glycosylation and ADAM/BACE2/proprotein convertase/γ-secretase proteolytic cleavages [23,24,25,26,27,28,29,30] and shuttled to stage I melanosomes. Mature PMEL fragments assemble into striated amyloid fibrils within stage II melanosomes, elongating the organelles and providing a scaffold for eumelanin deposition, driving melanosome maturation [24,25,31,32,33]. In this role, PMEL represents a protein of significant interest as a functional amyloid (discussed further in references [20,34]). Amyloid has historically been studied in relation to its toxicity and association with degenerative neuropathies such as Alzheimer’s and Parkinson’s disease. How the PMEL amyloid is tolerated within cells is therefore of major therapeutic interest [34,35].

**Figure 1:**
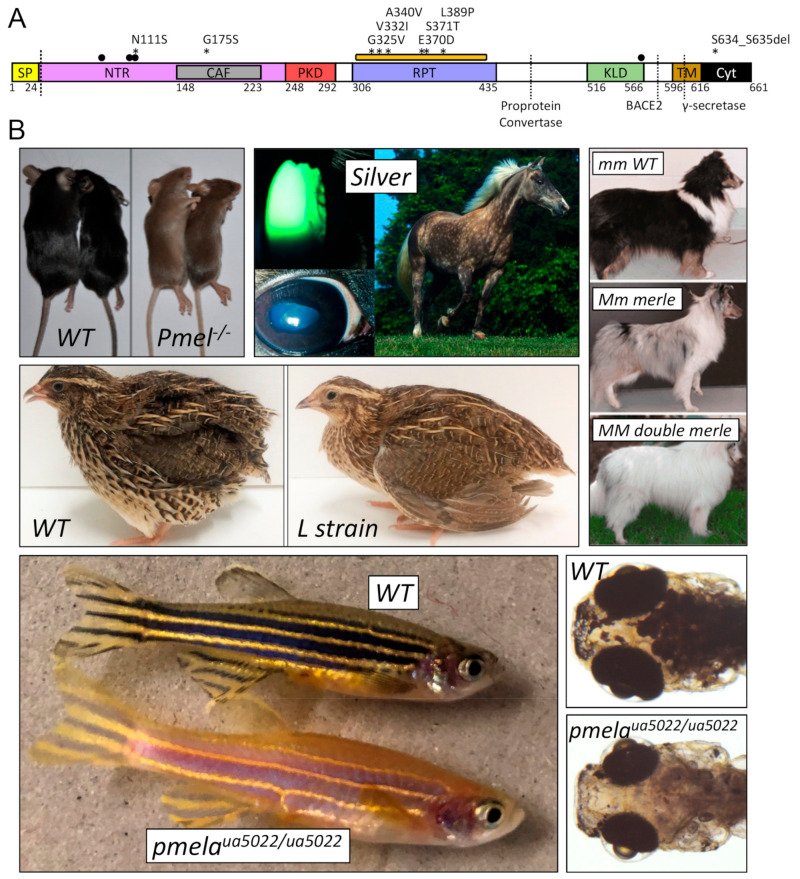
Human and animal mutations in *PMEL* result in hypopigmentation and ocular pathology. (**A**) Protein domain model for human premelanosome protein (PMEL) ENST00000548493.5 is shown, with residue positions determined from the alignments presented in Figures 7–9. Symbols at the top of the schematic indicate the location of: (1) human variants associated with pigment dispersion syndrome/pigmentary glaucoma [9] (asterisks), (2) N-linked glycosylation sites (black dots) and (3) the serine-/threonine-rich region of the RPT domain that is modified with O-linked glycosylation (orange rectangle). Various enzyme cleavage sites are depicted by dotted vertical lines. (**B**) Selected animal pigmentation and/or ocular defect images are reproduced below the model with permission. All phenotypes are caused by a mutation of the respective *PMEL* ortholog (see Table 1). From top-left to bottom-right: *Pmel*^-/-^ null mouse with a subtle dilution of coat and tail colors [40]; Silver Rocky Mountain Horse with cataracts and mitotic pupils, ectropion uvea, dyscoria, lens subluxation and a shiny white mane and tail, in conjunction with a slightly diluted body color with “silver” dapples [47]; coat color defects in merle (Mm) and double-merle (MM) Shetland Sheepdogs [49]; Japanese “L strain” quail with yellowish plumage [42] and *pmela*^ua5022/ua5022^ zebrafish with reduced global pigmentation and ocular anomalies that include enlarged anterior segments, microphthalmia and eyes that are more spherical in shape, suggestive of high intraocular pressure, a hallmark of glaucoma.

*PMEL* mutations have been selected in domesticated animals for at least a century [36] thanks to desirable changes in coats and plumage colors. However, it was not until the 1990s that molecular genetic analyses have identified the allele in *Pmel* responsible for the autosomal recessive, progressive greying fur of the Silver mouse [37,38]. Since then, a panoply of striking pigmentation patterns has been associated with mutations of the *pmel* gene (Figure 1). The majority of these represent recessive, loss-of-function mutations that give rise to hypopigmentation phenotypes [9,38,39,40,41,42]. However, dominant, missense mutations have also been described, suggestive of gain-of-function (GOF) pathology [16,17,43,44,45]. These dominantly inherited mutations sometimes cause more severe phenotypes, including ocular anterior segment dysgenesis (Figure 1) and deafness [46,47,48,49,50,51]. In humans, autosomal dominant pigmentary glaucoma is caused by missense *PMEL* mutations (Figure 1), providing a tantalizing suggestion that GOF mutations in a (usually) strictly regulated functional amyloid can lead to retinal neuropathy [9].

**Figure 2:**
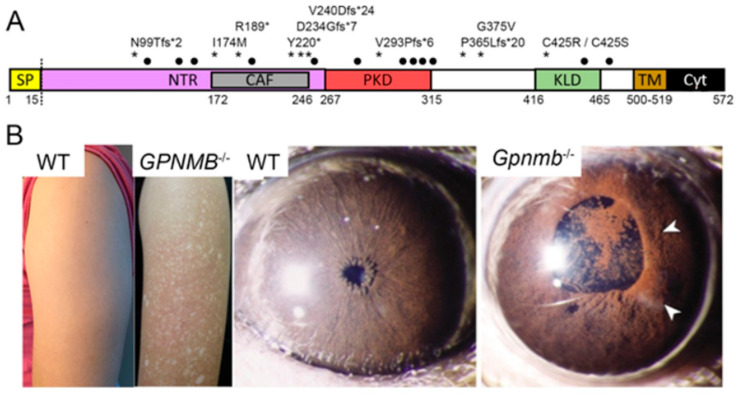
Human and animal mutations in *GPNMB* result in hypopigmented lesions, amyloidosis cutis dyschromica and ocular pathology. (**A**) Schematic representation of human GPNMB (NP_001005340.1). Asterisks indicate the locations of variants that cause amyloidosis cutis dyschromica (ACD) [18,63,64,65,66]. Locations of N-linked glycosylation sites (black dots) and the cleavage site (dotted vertical line) are depicted. (**B**) ACD presents with hypopigmented lesions (right, from reference [18]). A wild-type murine iris compared to the iris pigment dispersion (arrowheads) of a *Gpnmb*^-/-^ homozygote [8]. Images republished with permission.

Like PMEL, the GPNMB protein undergoes glycosylation and proteolytic cleavage to produce the mature peptide [52,53,54,55]. GPNMB also localizes to melanosomes [54,56,57], is transcriptionally regulated by the Melanocyte-Inducing Transcription Factor (MITF) [58,59,60,61] and, when mutated, can lead to hypopigmented lesions and pigmentary glaucoma in mice (Figure 2) [7,8,18,19]. Surprisingly, there has been no functional evidence of the amyloid-forming roles of GPNMB, perhaps owing to the post-transcriptional modification of its PKD domain [62], although disease presentation suggests a relationship between GPNMB and the amyloid. Human mutations of *GPNMB* cause recessive and semi-dominant amyloidosis cutis dyschromica [18,63,64,65,66], a condition characterized by amyloid deposition in the papillary dermis; remarkably, though, the GPNMB protein is not a constituent of amyloid deposits [18]. Furthermore, *GPNMB* is more broadly expressed than PMEL and has been implicated in a broad array of biological processes outside of pigmentation.

*GPNMB* is highly expressed in the nervous system, kidney nephrons, macrophages, dendritic cells and astrocytes [52,61,67,68,69,70]. A major role appears to be as a negative regulator of inflammation [71,72], and the breakdown of the ocular immune privilege is hypothesized to be the cause of *Gpnmb*-associated glaucoma in mice [73,74]. GPNMB is required for the differentiation of osteoblasts [58,67,75,76,77,78,79,80], and the protein localizes to the plasma membrane, as well as is cleaved in some cell types to act as a matricellular protein regulating extracellular matrix remodeling [53,54,55,58,81,82,83,84,85,86,87,88]. Most strikingly, the heightened expression of *GPNMB* has been associated with amyotrophic lateral sclerosis (ALS) [10,11], Parkinson’s disease [12,89,90] and Alzheimer’s Disease [91], all of which have pathogenic amyloids as major components of the disease progression. Perhaps counterintuitively, elevated levels of GPNMB are neuroprotective in Parkinson’s and ALS models, and the protein has not been described as a component of amyloid plaques [92,93,94].

Despite apparent differences in protein functions, the *GPNMB* and *PMEL* genes are putative paralogs that arose from an ancient common ancestor. In this paper, we synthesized the existing literature and performed new bioinformatic comparisons to help situate these (and future) comparisons of *PMEL* and *GPNMB* in the correct framework. We identified the enigmatic *TMEM130* as the most ancient paralog in the protein family, from which *PMEL* and *GPNMB* evolved in early craniate radiation. We then compared-and-contrasted their protein domains across a diversity of metazoans to identify the characteristic motifs unique to each gene. Of particular interest, the *PMEL*-specific repeat (RPT) domain, a key accessory domain to amyloid formation, was thoroughly analyzed across clade-representative species to interrogate the most highly conserved features of the domain. We hope that this work will stimulate interest in this important protein family, further elucidating the overlapping but divergent roles of GPNMB and PMEL and encouraging investigations into the functions of TMEM130.

## 2. Methods

### 2.1. Animal Ethics

Zebrafish were maintained and bred using typical husbandry methods and with the approval of the Animal Care and Use Committee at the University of Alberta (protocol AUP00000077). Zebrafish mutants *pmela^ua5022^* described previously as larvae [9] are assigned as ZFIN ID: ZDB-ALT-191119-1.

### 2.2. Identification of PKAT Family Homologs

PKAT (PKD- and KLD-Associated Transmembrane, see end of Section 3.2 for further explanation) homologs were identified via tblastn searches of the NCBI (https://blast.ncbi.nlm.nih.gov/Blast.cgi (accessed on 19 March 2021)) and ENSEMBL (https://uswest.ensembl.org/Multi/Tools/Blast?db=core (accessed on 19 March 2021)) genome assembly databases. For *E. muelleri*, the BLAST search was performed at https://spaces.facsci.ualberta.ca/ephybase/ (accessed on 19 March 2021). The query sequence was human TMEM130, GPNMB or the PMEL PKD domain. All default parameters were maintained, including BLOSUM62 and an E-value cut-off of 0.05. For predicted/uncharacterized loci, a reciprocal blastp to the human assembly was performed. Percentage homology between paralogs was reported as the sequence identity/sequence length calculated after Clustal Omega alignment.

### 2.3. Annotating PKAT Protein Domain Architecture and Intron–Exon Architecture

Human TMEM130 (ENST00000416379.6), GPNMB (NP_001005340.1) and PMEL (ENST00000548493.5) protein sequences were analyzed with the InterPro Scan web client (https://www.ebi.ac.uk/interpro/search/sequence (accessed on 19 March 2021)) [95] to receive domain predictions. Schematic representations of these domains were created in CorelDRAW X7. Exon length was determined by uploading genomic DNA sequences of each gene into Geneious software and exporting the exon length information. Schematics representing the protein domain boundaries were created in CorelDRAW X7.

### 2.4. Production of Cladogram/Phylograms

The clade names and branching structure reported in Section 3.3 were derived from the NCBI Taxonomy Browser, building a phylip-formatted tree with selected taxa at https://www.ncbi.nlm.nih.gov/Taxonomy/CommonTree/wwwcmt.cgi (accessed on 19 March 2021). The phylogram reported in Section 3.4 was created using clade-representative protein sequences. Sequences were uploaded to MEGA X, and phylogeny was inferred using the Maximum Likelihood method and Whelan and Goldman + Frequency model. One thousand bootstrap iterations were performed, and Trichoplax tmem130 was chosen as the outgroup. Color of the monophyletic groups was added in Photoshop v22.1.

### 2.5. Synteny Analysis

One hundred genes upstream and downstream (or until the end of the chromosome) of the lamprey PKAT outparalogs were downloaded from the NCBI genome assembly (kPetMar1.pri). The gene identity tracks were then compared against the human Ch7 and Ch12 (GRCh38.p13), chicken Ch2 and Ch33 (GRCg6a) and skate Ch2 and Ch46 (sAmbRad1.pri). Genes shared with any of the other species were used as evidence of paralogous regions. The schematic representation figure was produced in Photoshop v22.1.

### 2.6. Functional Domain Alignments

Representative protein sequences for each gene in each major clade were chosen from the annotated and curated organismal lists at Ensembl (https://uswest.ensembl.org/index.html (accessed on 19 March 2021)) or NCBI (https://www.ncbi.nlm.nih.gov/ (accessed on 19 March 2021)) where possible. For incompletely-annotated genes, BLASTp and tBLASTn [96] searches of the NCBI database (https://blast.ncbi.nlm.nih.gov/Blast.cgi (accessed on 19 March 2021)) were performed to identify representative gene entries or unannotated gene fragments in whole-genome shotgun contigs. Protein sequences were aligned in Geneious Prime 2020.1.2, created by Biomatters (https://www.geneious.com (accessed on 19 March 2021)), using the MUSCLE algorithm and manual editing.

### 2.7. RPT Domain Identification and Comparison

Rapid Automated Detection of Repeats (RADAR) (https://www.ebi.ac.uk/Tools/pfa/radar/ (accessed on 19 March 2021)) [97,98] was used to identify repetitive regions in the premelanosome (*Pmel*) protein amino acid sequences prior to aligning repeats via similarity by hand. The corresponding cDNA of each protein repeat was aligned to correspond with the amino acids, polarity color coding was selected and consensus sequences and sequence logos were made in Geneious Prime.

## 3. Results and Discussion

### 3.1. Mutations of PMEL or GPNMB Cause an Overlapping Spectrum of Pathologies but Highlight the Disparate Functional Roles of Each Protein

Many mutations have been described in the *PMEL* gene from a variety of animals, owing to the striking pigmentary changes that result (Figure 1). Correlating genotypes to phenotypes reveals that missense mutations in *PMEL* homologs typically exhibit a dominant inheritance pattern (Table 1 and the references therein). For example, the dominant inheritance of the pigment phenotypes in white chicken, Charolais cattle and Silver dapple horses are caused by missense mutations in *PMEL*. This contrasts the recessive pigmentation phenotypes in the Silver/*Pmel*^-/-^ mouse and two alleles of zebrafish, which harbor nonsense mutations (Table 1). This correlation is also apparent in human dominantly inherited pigmentary glaucoma associated with missense mutations in *PMEL* (Table 2 and the references therein). The exceptions to this trend include a variable expansion of the SINE intron insertion within Merle dogs that is a dominantly inherited nonsense mutation. This overall pattern suggests that nonsense mutations in *PMEL* homologs are often better-tolerated and require two mutant alleles for detectable phenotypes. However, the phenotype–genotype correlation for pigmentation defects is not retained across the ocular phenotypes associated with *PMEL* mutation, which have been described in zebrafish (recessive), horses (recessive), dogs (recessive) and humans (dominant). 

In contrast, the human disease amyloidosis cutis dyschromica (Figure 2) is caused by *GPNMB* mutations with both recessive and semi-dominant inheritance patterns described (Table 3 and the references therein). Causal mutations can be either nonsense or missense, and the symptoms include generalized hypopigmented skin mottled with hyperpigmented macules [18]. While no detectable phenotype has been described in zebrafish *gpnmb* mutants, murine *Gpnmb*, *Tryp1* double-homozygotes develop pigment dispersion, pigmentary glaucoma and iris atrophy (Table 4) without reported dermal amyloidosis [8,74]. These murine phenotypes highlight an intriguing overlap between *Gpnmb* and *Pmel*, as the mutations in each can result in pigmentary and ocular defects.

### 3.2. TMEM130, GPNMB and PMEL Form a Protein Family and Share a Common Protein Domain Architecture

*GPNMB* and *PMEL* have long been recognized as outparalogs (genes arising from an ancient duplication event (for review, see reference [99]) and studied together as a gene family. However, *TMEM130* is also a recognized member of the PANTHER melanocyte protein PMEL-17-related (PTHR11861) family (http://www.pantherdb.org (accessed on 19 March 2021)) but remains poorly studied. If *TMEM130* does indeed represent a third member of the gene family, then comparisons of the three genes could provide insight as to how *PMEL* and *GPNMB* evolved different functions. 

**Figure 3:**
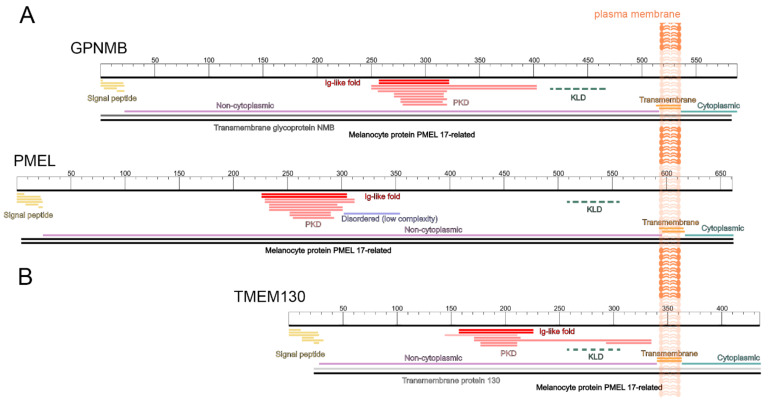
GPNMB, PMEL and TMEM130 form a family of related proteins with a homologous domain architecture. Schematic representation of the human GPNMB (ENST00000647578.1) and PMEL (ENST00000548493.5) proteins (**A**), and TMEM130 (ENST00000416379.6) (**B**) transmembrane proteins annotated with their predicted function domains. Each protein is represented by a segmented white box displaying the relative amino acid length: 588, 661 and 435 residues, respectively. Below the protein schematic, the InterPro Scan domain predictions are denoted by colored lines to represent the signal peptide (yellow), Ig-like fold (red), polycystic kidney disease domain (PKD, pink), non-cytoplasmic region (purple), transmembrane domain (orange) and cytoplasmic region (green). PMEL was predicted to possess a disordered (low complexity) region (blue). The homology of these sequences to GPNMB (dark grey), PMEL (black) and TMEM130 (light grey) was also flagged. The Kringle-like domain (KLD, dotted green line) was not detected by InterPro Scan but was identified by the sequence alignment. This order and arrangement of the functional domain is characteristic of the PKD- and KLD-Associated Transmembrane (PKAT) family.

Despite being predicted homologs, the MUSCLE alignment of the full-length human sequences (Tables S1 and S2) demonstrated the limited sequence identity of *PMEL* to the other members at either the cDNA level (38.7% vs. *TMEM130* and 33.2% vs. *GPNMB*) or the protein level (13.7% vs. TMEM130 and 24.9% vs. GPNMB). We therefore opted to investigate the similarities at the level of the shared domain architecture. The sequences for human TMEM130 (ENST00000416379.6), GPNMB (ENST00000647578.1) and PMEL (ENST00000548493.5) proteins were run through InterPro Scan to identify the functional domains based on the database signatures [95]. As expected, the GPNMB and PMEL proteins displayed a conserved protein domain architecture: (1) an N-terminal secretory pathway targeting signal (signal peptide, SP), (2) a large non-cytoplasmic (luminal) region containing an immunoglobulin-like polycystic kidney domain (PKD) and (3) a single Type I transmembrane domain with a short, C-terminal cytosolic domain (Figure 3A and Table S3). 

The InterPro Scan analysis does not annotate the highly conserved Kringle-like domain (KLD) that has been previously characterized in *GPNMB* and *PMEL* [100,101]; this domain, (named after the triple-loop fold Kringle domain) lies downstream of the PKD (Figure 3A dashed line) and contains six highly conserved cysteine residues that are critical for forming the disulphide bonds of mature PMEL dimers [100]. However, the InterPro Scan analysis does identify a disordered (low complexity) region unique to PMEL. This region corresponds to the enigmatic PMEL repeat domain (RPT), which is sufficient to form fibrils outside of the normal physiological conditions [21,102,103] but is dispensable for amyloid formation in vivo [22,104,105]. Instead, the most recent data suggests that the RPT is an essential accessory domain required for physiological amyloid packing [22]; this is consistent with the absence of a RPT domain in GPNMB, which does not contribute to melanosome amyloid [62].

An analysis of the human TMEM130 domain architecture revealed that, despite being considerably shorter than the other two family members (435 residues vs. 588 GPNMB and 661 PMEL), TMEM130 has a strikingly similar domain architecture (Figure 3B and Table S3). The signal peptide, PKD and transmembrane domains were present and conserved in order with the other two proteins. Furthermore, a KLD was resolved via multiple sequence alignment (see Section 3.6). Despite lacking identifiable homology in the region between the SP and PKD domains, this analysis strongly supports the inclusion of TMEM130 as a member of the same protein family. Consistently, all three proteins were identified as “melanocyte protein PMEL-17- related family members”. This nomenclature is based upon the previous naming convention of PMEL (PMEL-17) and is outdated. We therefore propose the use of “PKD- and KLD-Associated Transmembrane (PKAT) protein family” for this interesting protein family. This name reflects the highly conserved domain architecture of the three human gene members and remains inclusive of species-specific paralogs (inparalogs) that arise during gene duplication events (see Section 3.3).

### 3.3. The PKAT Family Genes Are Distantly Related Paralogs and TMEM130 Represents the Most Ancient Homolog

**Figure 4:**
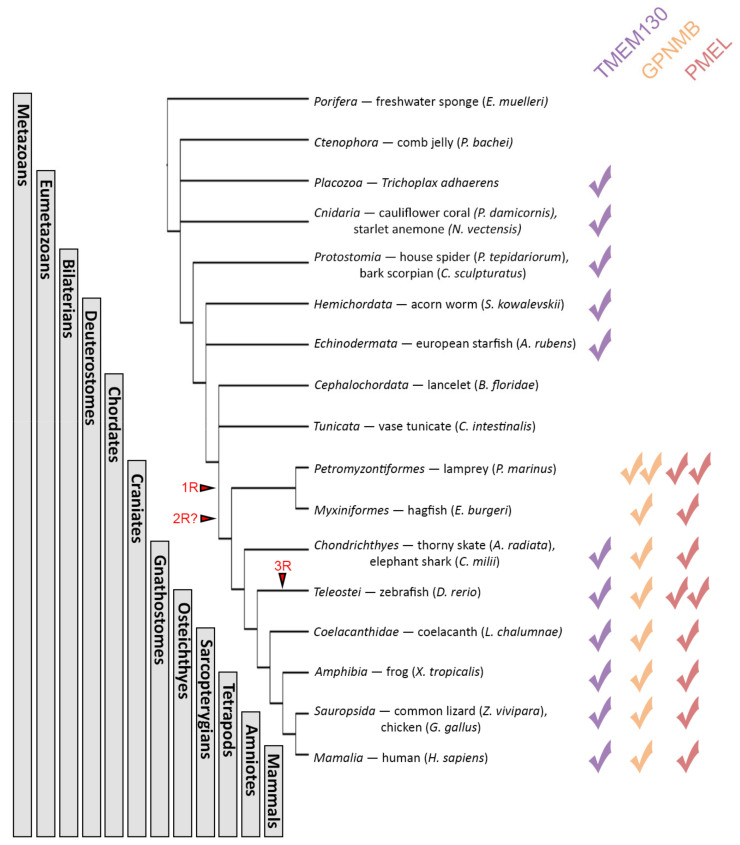
*TMEM130* is the most ancient member of the PKAT family in the basal metazoa, with GPNMB and PMEL originating prior to the craniate radiation. Schematic representation of the major animal taxa and cladogram demonstrating their evolutionary relationship and where whole-genome duplication events occurred (1R, 2R and 3R). Check marks demonstrate which of the paralogous genes are found within the species inside these taxa. The presence of two check marks of the same colour indicates that two paralogs of that gene are found in that species.

The conservation of the domain architecture between all three PKAT members suggests that these genes are either related by descent (homologs) or arose via convergent evolution. To investigate if there is support for homology, we first investigated when each gene arose. Using the human PKD domains, we can identify hundreds of potential homologs from the ENSEMBL and NCBI databases using translated nucleotide (tblastn) searches. A selection of these hits has been used as representatives of major animal clades and was included in all subsequent analyses (Table S4, Supplementary Materials). The first evidence of putative *tmem130* orthologs was discovered in Placozoa (*T. adhaerens*) and *Cnidaria* (*P. damicornis* and *N. vectensis*), but no regions of significant sequence identity can be detected in the early metazoan genome assemblies of *Porifera* (*E. mulleri*) and *Ctenophora* (*P. bachei*) [106,107]. In more derived species, including the *Protostomia*, *Hemichordata* and *Echinodermata*, *tmem130* orthologs can also be identified (Figure 4); however, no *gpnmb* or *pmel* orthologs were identified in any of the invertebrate clades examined. These data suggest that *tmem130* is the most ancient of the PKAT family genes and originated in early eumetazoans prior to the divergence of *Cnidaria*, Placozoa and *Ctenophora.*

Orthologs of all three PKAT family members were identified in cartilaginous fish (*A. radiata* and *C. milli*), and all subsequent *Gnathostomes* that were analyzed (Figure 4). Assuming the PKAT genes are homologs then this pattern suggests that two rounds of *TMEM130* duplication followed by neo- or sub-functionalization [108,109] gave rise to vertebrate *TMEM130*, *GPNMB* and *PMEL*, with the final of the four paralogs being lost. This proposal is consistent with the two rounds of whole-genome duplications (1R and 2R, Figure 4) that occurred during early vertebrate evolution [110,111,112]. While the exact timing of the 2R duplication is still contested [113,114,115,116,117], the inheritance pattern of the PKAT gene family favors the 2R duplication occurring prior to the divergence of the Cyclostomata: *gpnmb* and *pmel* orthologs discovered in lamprey (*P. marinus*) and hagfish (*E. burgeri*). Intriguingly, no evidence for the *tmem130* orthologs were discovered in the early *Chordates* lancelet (*B. belcheri*) or tunicate (*C. intestinalis*), nor in the Cyclostomes, suggesting an independent *tmem130* gene loss in these species [118] or incomplete genome sequences that prevent orthologs from being identified. The *tmem130* orthologs were also absent from several protostome species assemblies that we probed (e.g., *C. elegans* WBcel235 and *Octopus vulgaris* ASM395772v1), despite orthologs existing in other members of the clade. In conjunction with the absence of *tmem130* in basal chordates, these data suggest that *TMEM130* has been lost independently multiple times throughout animal evolution. Conversely, duplication of the PKAT genes was also apparent. Two *gpnmb* and two *pmel* orthologs were discovered in the lamprey, presumably via a segmental duplication similar to that which gave rise to the six lamprey hox clusters compared to the four found in most tetrapods [119]. Furthermore, zebrafish (and other teleosts) possess two recent *PMEL* inparalogs (*pmela* and *pmelb*) but only one copy of the other PKAT genes, likely because of the teleost-specific genome duplication event (3R, Figure 4) and subsequent rapid gene loss [120,121,122,123]. Taken together, these data support a paralogous relationship between the PKAT family genes and demonstrate that *TMEM130* is the most ancient, eumetazoan gene from which the other two genes evolved. It has not escaped our notice that the emergence of *GPNMB* and *PMEL* also coincides with the evolution of the complex, camera-style vertebrate eye. Considering the ocular phenotypes that arise when these genes are mutated, it seems plausible that these two events are related.

**Figure 5:**
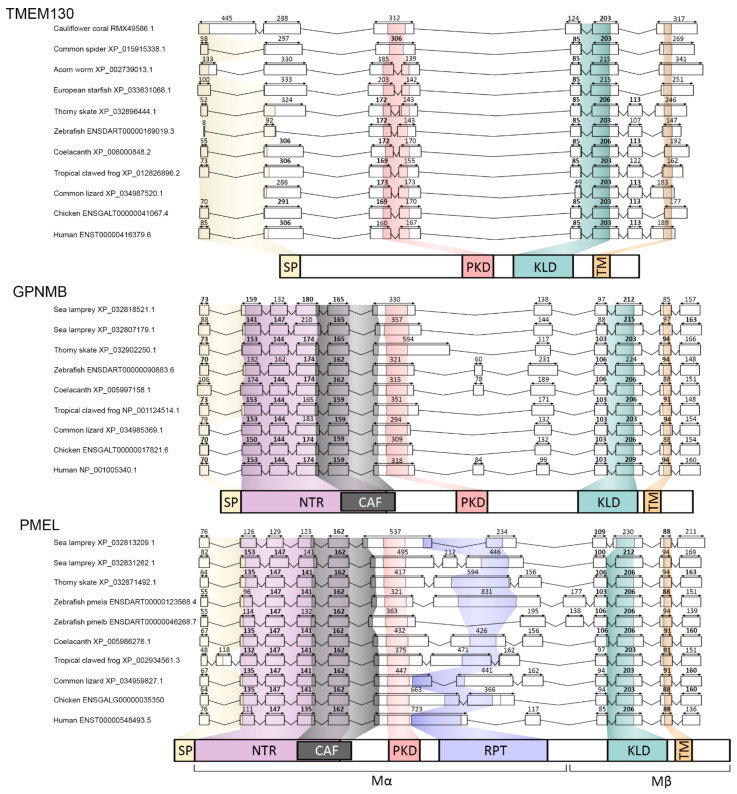
The intron-exon architecture supports a common ancestry for the PKAT family genes. Schematic representation of gene family orthologs showing exons (boxes, to scale) and separated by introns (lines, not to scale). The nucleotides coding for different protein domains are colour coded: signal peptide (yellow), N-terminal region (NTR; purple), core amyloid fragment (CAF; black) polycystic kidney domain (PKD; red), repeat domain (RPT; blue), kringle-like domain (KLD; green), transmembrane domain (TM; orange). Numbers above the exons denote exon length in basepairs, bold donates when exon lengths are shared between >3 orthologs (±5%).

To further interrogate the paralogous relationship of the PKAT genes, we next examined the intron–exon structure of the clade-representative genes. The exon length (bps) from the PKAT genomic DNA sequences was correlated with the functional domains that they encoded. When displayed graphically, several features were apparent that supported a shared origin (Figure 5). A signal peptide domain was detected in all the sequences examined and was encoded by the first two exons of the gene. In 27/31 genes, the majority of the SP was encoded within an initial, small (<110 bp) exon. The Kringle-like domain was encoded by two exons in 30/31 genes examined, and the exon lengths were conserved by ± 5% in most of the clade representatives: *TMEM130* 85 bp (9/11) and 203 bp (9/11), *GPNMB* 103 bp (7/10) and 206 bp (7/10) and PMEL 106 bp (6/10) and 206 bp (9/10). This analysis also supported an ancient homology, because the close similarities of the exon sizes between these three genes were consistent with them arising from a common origin. 

Interestingly, this comparison of gene architectures revealed differences between the genes that speak to the evolution and divergence of the gene family. Firstly, the PKD domain was encoded by two exons in 9/11 of the *TMEM130* genes examined but within a single exon in *GPNMB* and *PMEL* (Figure 5). This suggests that there was a loss of the intron from the PKD after the 1R genome duplication and prior to the divergence of *GPNMB* and *PMEL* as novel genes. Furthermore, there were multiple exons introduced upstream of the PKD in the *GPNMB* and *PMEL* genes that were absent from *TMEM130*. These exons (ex2-5) showed particularly high conservations ±5% of the exon length in both *GPNMB* (153 bp (7/10), 144 bp (6/10), 174 bp (6/10) and 159 bp (5/10)) and *PMEL* (135 bp (6/10), 147 bp (9/10), 141 bp (6/10) and 16 2bp (10/10)), respectively, supportive of a shared origin. In *PMEL*, these exons encoded for the N-terminal region and core amyloid fragment, which was critical for the formation of amyloid filaments [1]. It was therefore surprising that the exon length was conserved in this region in GPNMB that does not form amyloids; however, this apparent contradiction may be due to a lack of protein residue conservation between the two. Finally, the RPT domain, which is found only in *PMEL* orthologs, was encoded within either one or two exons in the species examined without any discernable patterns between the species.

### 3.4. TMEM130 Is a Sister Group of Both GPNMB and PMEL

**Figure 6:**
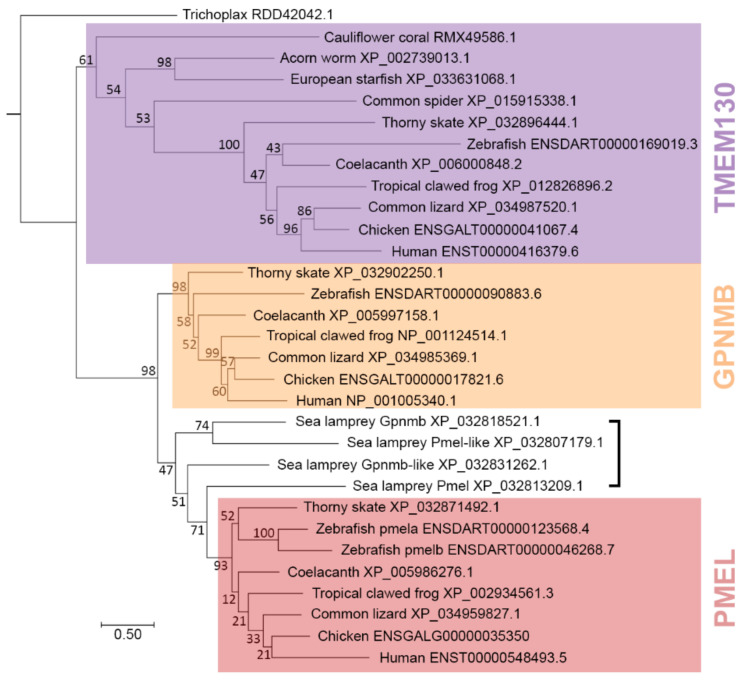
Phylogenetic tree representing the outparalog TMEM130, GPNMB and PMEL protein sequences separated by speciation events. Phylogeny was inferred by the Maximum Likelihood method and Whelan and Goldman + Frequency model. The BS ×1000 and percentage of trees in which the associated taxa clustered together are shown next to the branches. Branch lengths represent the substitutions per site.

With evidence of a paralogous relationship between the PKAT family genes, we next produced a Maximum Likelihood phylogenetic tree based on the whole-protein sequence alignment of the clade representative sequences. TMEM130 was the most ancient paralog detected (Figure 4) and was selected as an outgroup for tree construction, and the *Trichoplax* sequence was used as the most basal PKAT sequence that was identified. Within our model, there are three strongly supported monophyletic groups that correlated to TMEM130, GPNMB and PMEL (Figure 6). In our phylogram, TMEM130 formed a sister group with both GPNMB and PMEL. 

Intriguingly, sea lamprey possesses four inparalogs: *pmel*, *pmel-like*, *gpnmb* and *gpnmb-like* but no putative *tmem130* ortholog. These inparalogs cannot confidently be separated into the three clades and, instead, form a separate, paraphyletic group of their own (Figure 6, bracket). Unexpectedly, Gpnmb and Pmel-like are predicted to be more closely related to each other, whereas pmel and gpnmb-like are separately more closely related to each other, suggesting that these inparalogs may be incorrectly annotated. To test whether lamprey *gpnmb-like* (XP_032831262.1) and *pmel-like* (XP_032807179.1) were named incorrectly, we examined the chromosomal synteny of up to 100 genes upstream and downstream of the lamprey PKAT genes, making comparisons to the homologous chromosomal regions (paralogons) of both GPNMB and PMEL in three Gnathostomes: human, chicken and thorny skate (supplemental dataset). All four lamprey chromosomal regions have syntenic genes in common with *Gnathostome GPNMB* and *PMEL*; surprisingly, for each lamprey PKAT gene, there are more neighboring genes in common with the homologous *Gnathostome* GPNMB region than with the PMEL regions (Figure S1, Supplementary Materials). Lamprey *gpnmb* and *pmel-like* possess the strongest signals, each with a higher ratio of genes syntenic with Gnathostome *GPNMB* than with *PMEL* (47/3 = 15.7 and 33/6 = 5.5, respectively). Conversely, lamprey *pmel* and *gpnmb-like* have a lower ratio of genes syntenic with Gnathostome *GPNMB* than with *PMEL* (22/16 = 1.4 and 25/10 = 2.5, respectively). Since these analyses demonstrate that the lamprey paralogons have mixtures of homologous genes relative to both the *GPNMB* and *PMEL* chromosomal regions (Table S5, Supplementary Materials), it was therefore not possible to conclude what gene names ultimately should be applied to the lamprey inparalogs *pmel-like* and *gpnmb*-like via synteny analysis alone. However, Gpnmb-like does appear to possess an RPT domain (discussed in detail below), suggesting that it may be the canonical inparalog of *pmel*.

### 3.5. The Amino-Terminal Region of PKAT Proteins Show Conservation of a Signal Peptide and the Core Amyloid Fragment in PMEL and GPNMB

**Figure 7:**
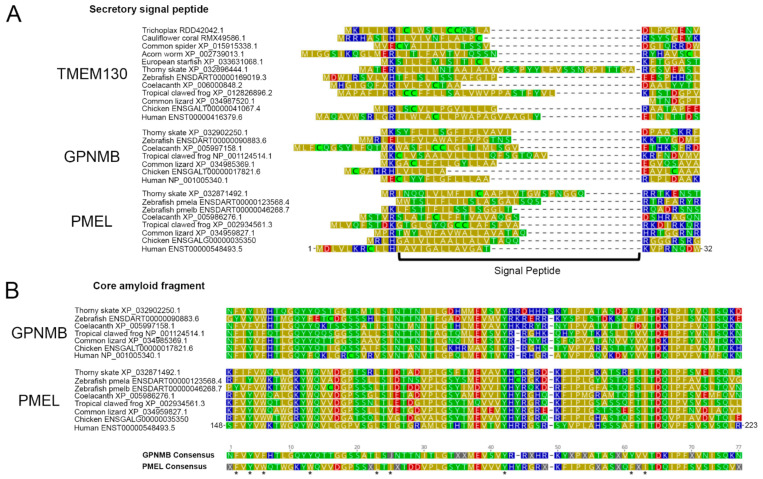
The N-terminal region of the PKAT paralogs shows conservation of a signal peptide in all family members and conservation of the core amyloid fragment in GPNMB. The domains are shown for human PMEL ENST00000548493.5, with residue positions shown, aligned to paralogous sequences from the representative species. The residues are color-coded for the polarity of their amino acid side chains (yellow, nonpolar; green, uncharged polar; red, acidic; blue, basic). (**A**) The representative sequences of TMEM130, GPNMB and PMEL contain a predicted secretory signal peptide, rich in nonpolar residues. (**B**) GPNMB and PMEL paralogs show a striking conservation throughout their N-termini, especially in the PMEL Core Amyloid Fragment [126], where the “essential residues” are labeled with asterisks (*). J = leucine or isoleucine.

Despite the poor overall sequence conservation between the PKAT family proteins, several functional domains have been described as being well-conserved between PMEL and GPNMB [1]. The conservation of functional domains is suggestive of a common function within the cell; however, as discussed in the introduction, there has been a dearth of studies that demonstrate that the two proteins function within the same pathway. Furthermore, nothing has been published about the role of TMEM130 in the cell. To interrogate the conserved and divergent domains of the family, we performed multiple sequence alignments of clade-representative sequences. Firstly, the presumptive signal peptides (SPs) from our clade representatives display little sequence homology, as expected for a nonstructural determinant of the protein function [124]. Instead, we observed a stretch of eight or more amino acids with uncharged side chains, which were also rich in nonpolar side chains [125]. We determined that most of the outparalogs have a basic or acidic charged residue within 1–13 positions after the start methionine, followed by an easily detected SP of 7–33 residues long, before another basic or acidic residue appears (Figure 7A). The SP is cleaved from the mature protein in the ER once it has been translocated into the secretory pathway. Our alignment also revealed that there may be errors in the gene annotation of common lizard *tmem130*, where the start methionine of the sequence aligns downstream of the conserved position of SPs; correspondingly, its gene model lacks the “exon 1” that is annotated for all the other *TMEM130*s.

It has long been known that the N-terminal regions, following the SP of PMEL and GPNMB, share considerable sequence homology [1]. However, some 12 years after this discovery, the essential residues necessary for the formation of PMEL amyloids were uncovered via an unbiased alanine-scanning screen [126]. These residues overlap the PMEL N-terminal region and constitute the core amyloid fragment (CAF). By aligning the GPNMB and PMEL orthologs separately to create a GPNMB consensus and PMEL consensus, we demonstrated that there was considerable sequence identity conservation within these two regions (Figure 7B). Several key aromatic residues were shown to be critical for PMEL amyloid formation, and the mutagenesis of these residues prevented amyloid formation [126]. Interestingly all of these residues were biochemically conserved (but not identical) in GPNMB (Figure 7B, asterisks), despite no known amyloid-forming potential. The CAF sequence was not detectable in TMEM130, and there was no significant region of homology detected upstream of the PKD (data not shown).

### 3.6. Multiple Carboxy-Terminus Domains and Functional Motifs Are Conserved in the PKAT Family

**Figure 8:**
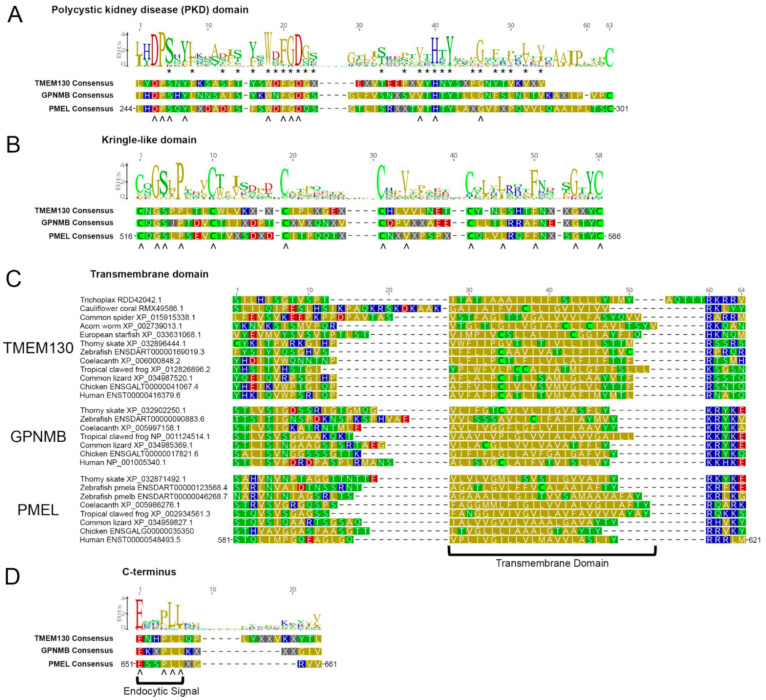
Multiple domains and functional motifs of the C-terminal to the Core Amyloid Fragment are conserved in the PKAT family. Sequence logos showing the relative abundance of each residue found at a given position were derived from alignments of paralogous sequences from the 27 clade-representative species (see Table S4, Supplementary Materials). The PMEL residue positions are shown for human PMEL ENST00000548493.5. The residues are color-coded for the polarity of their amino acid side chains (yellow, nonpolar; green, uncharged polar; red, acidic; blue, basic). Below the alignments, consensus sequences are shown for each separate orthologous gene group. Carets (^) denote residues that match each consensus, thus identifying the key residues conserved in the PKAT gene family (TMEM130, GPNMB and PMEL). (**A**) In the region C-terminal to the Core Amyloid Fragment of PMEL, many amino acid residues, marked with asterisks (*), are well-conserved relative to the sequence logo for the PKD domain (PROSITE PDOC50093). (**B**) In the C-terminal region to the RPT domain of PMEL, termed the Kringle-like domain, six cysteines are perfectly conserved for all the GPNMB and PMEL sequences and mostly conserved for the TMEM130 sequences. (**C**) In the C-terminal region to the Kringle-like domains, all of the PKAT gene family paralogs contain a single-pass transmembrane domain, rich in nonpolar residues. (**D**) At their C-termini, the PKAT gene family paralogs show the conservation of an endocytic signal matching the motif [DE]XXXL[LI].

The PKD domain is named after the polycystic kidney disease-associated gene *PKD1* [127], which possesses 17 PKD domains (P98161—www.uniprot.org). PKD domains have been described in several membrane-anchored glycoproteins (e.g., PKD1, PKD1L1 and SORCS1-3) and are often expected to play a role in protein–protein/protein–matrix interactions [128]. By analyzing the PKAT proteins, we found that many of the residues were well-conserved with the archetypal PKD domain (Figure 8A, carets), including the core WDFGDGS motif [129]. However, we also determined that key residues in the PKAT PKD domain were distinct from other PKD domains i.e., the [LI][HY]DP motif in positions 244–247 (Figure 8A). These commonalities could be a useful motif for assigning newly discovered sequences to the family and in predicting which residues are most critical for protein structure and function. Only GPNMBs and PMELs have a conserved cysteine at the end of the PKD domain (position 301 in Figure 8A), which corresponds to PMEL Cys301 and has been shown to be critical for protein dimerization [100]. The presence of this cysteine in GPNMBs suggests that GPNMB also dimerizes in the same fashion as PMEL.

Next, we generated a sequence logo for the Kringle-like domains (KLDs) of all 27 clade-representative sequences and generated separate consensuses for each ortholog (Figure 8b). The six cysteine residues were perfectly conserved in both GPNMB and PMEL and highly conserved in the TMEM130 sequences (Figure S2, Supplementary Materials); the inter-amino acid spacing of the KLDs was also well-conserved in all outparalogs. The KLD cysteine residues were shown to be required *PMEL* dimerization [100], strengthening the evidence for GPNMB dimerization. The C-terminal to the KLD type 1 transmembrane domains of the outparalogs were composed primarily of nonpolar amino acid side chains (Figure 8C). There was poor sequence conservation overall for these residues, suggesting that the TM domain was conserved merely for membrane localization of the proteins and not for other functions. 

Finally, all three family members have conservation of a C-terminal dileucine-based endocytic signal motif of [DE]XXXL[LI] sequence (Figure 8D) [130]. A notable exception is zebrafish Pmelb that lacks the two leucines. The endocytic signal is necessary to prevent the accumulation of PMEL at the plasma membrane [39], in turn allowing its internalization into the multivesicular bodies that become melanosomes. One could predict that zebrafish Pmelb might therefore be present only at the plasma membrane or be secreted and thus not accumulate in melanosomes. Alternatively, Pmelb could be recruited to melanosomes via some other process. Likewise, the dileucine signal in quail GPNMB/QNR-71 mediates its intracellular transport away from the cell membrane to localize the protein in endosomal/premelanosomal compartments [131]. TMEM130 in basal metazoans (coral and Trichoplax) also have poor conservation of the endocytic signal motif; this may be due to (1) the relatively low-quality genomic sequence/annotation of these gene entries, (2) that these organisms have different constraints on endocytosis or (3) different functions of TMEM130 in these species.

The strong conservation between the PKAT family members may be considered surprising, if one interprets the phenotypic effects as relatively minor in these loss of function mutations across a variety of species (Figure 1 and Figure 2; [40]). Pigmentation may be very important to survival, as it has impacts on camouflage, social interactions, protection from damaging electromagnetic radiation and mating. Moreover, any subtle deficits in vision or neuroprotection pathways would be selected against. Perhaps these and other potential functions provide enough selective pressure on this gene family to support strong purifying conservation. Alternatively, if mutations are likely to create a dominant and undesirable phenotype (akin to what is described with chicken pmel mutations), there would be a selection against them in a natural population [16,45]. Additionally, the PKAT family could provide some redundancy for an important developmental role that we have been unable to detect. Continuing studying into the functions of the PKAT family members—in particular, the more distantly related and ancient TMEM130 or concurrent mutations of all PKAT genes within an animal—may reveal why they have retained shared features over evolutionary time.

### 3.7. The PMEL RPT Domain Sequence Is Highly Divergent between Species but Considerably Conserved within Repeat Units of Most Species

The repeat domain (RPT) is a low complexity region unique to PMEL, and in mammals, it contains a variable number of proline/serine/threonine-rich imperfect repeats [103]. The RPT domain is controversial with respect to its role in PMEL amyloid formation, having originally been shown to be sufficient for fibril formation in vitro [102,132] but also dispensable for the formation of fibrils containing the N-terminal region and PKD domains [104,105]. Recent studies have instead demonstrated that the RPT domain has an accessory role in amyloid formation, at least in the few species studied to date. The PMEL RPT domain undergoes the heavy O-glycosylation of Thr/Ser residues that are required to sustain the sheet-like architecture of melanosomal amyloids. Deletion of the RPT domain or pharmacological inhibition of O-glycosylation causes amyloid collapse. Conversely, replacement of the RPT domain with a region of the MUC2 gene (which is also highly O-glycosylated) can rescue the fibril architecture [22]. 

**Figure 9:**
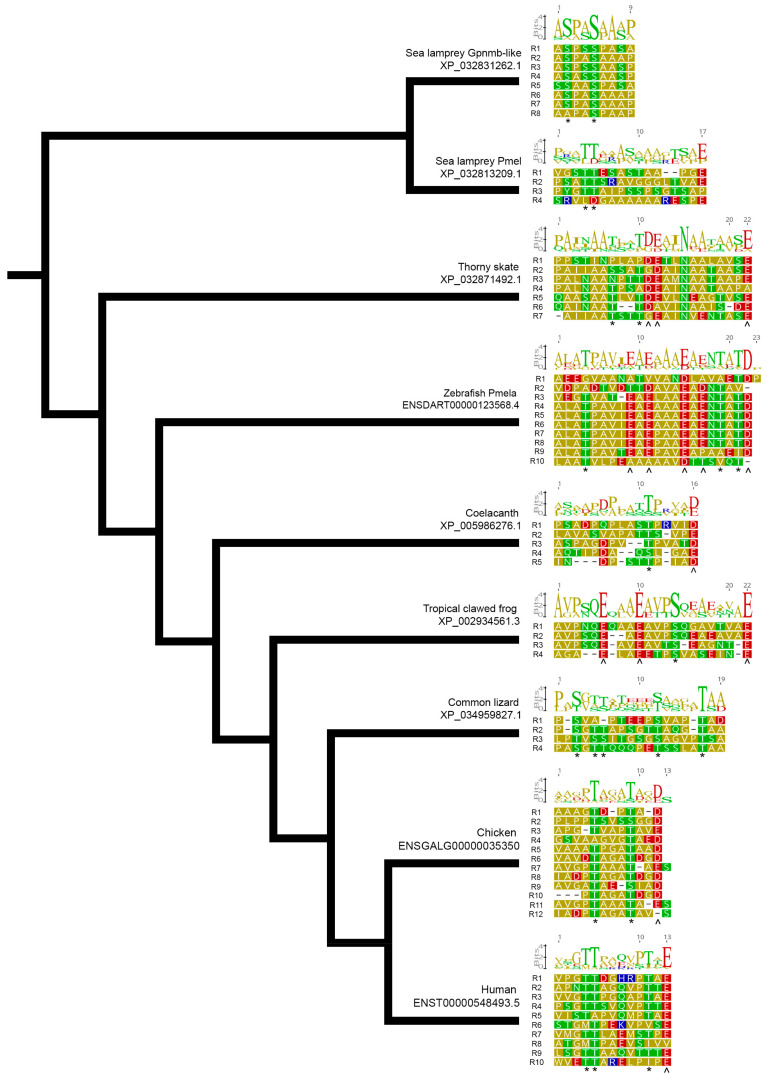
The PMEL repeat domain varies in size and amino acid composition, but T/S ladders and D/E ladders are a frequent feature. Alignments of individual PMEL repeat units of clade-representative organisms displayed in a cladogram. Sequence logos above the raw sequence represent the degree of sequence identity within a species. Conserved T/S ladders * and conserved D/E ladders ^. Residues are color-coded for the polarity of their amino acid side chains (yellow, nonpolar; green, uncharged polar; red, acidic; blue, basic).

To investigate the evolutionary relationship between orthologous PMEL RPT domains, we used RADAR predictions [97,133] and manually curated these to predict the RPT domain sequences of each PMEL protein (Figure 9 and Figures S3–S6, Supplementary Materials). Our results highlighted the high variability of the RPT domain in terms of the repeat unit length and repeat unit number between clade-representative organisms (Figure 9, c.f., thorny skate has seven 22-mer repeats vs. human has ten 13-mer repeats). These data also highlight the difficulty in accurately predicting the RPT domain sequence, since some organisms, like the common lizard, have a dearth of repeating residues per unit yet retain a similar chemical composition. Furthermore, even within a clade, there is much variability; for example, 11/11 mammalian proteins examined have 13-mer repeats, but these range from five repeat units in the Tasmanian devil to 16 units in sperm whale PMEL (Figure S3); in fish, the repeat unit length is highly variable, as exemplified by the difference between cod (11-mer length) and Pachon cavefish (26-mer length) (Figure S5). 

The primary sequences of individual repeats are also drastically different between species. The human RPT contains two motifs that we noted are highly conserved amongst the repeats: GTT and PTXE; in contrast, the chicken RPT domain has a much more divergent TXXXTXX[DE] motif, despite also being a similar 12-mer-repeating unit. These striking differences in sequences are especially remarkable considering that the chicken RPT domain is sufficient to rescue the fibrilogenesis defects of human PMEL lacking a RPT domain, while the zebrafish RPT domain can provide a partial rescue [22]. These data suggest that, despite huge variations in RPT domain evolution, this region has a conserved protein function. 

Despite the extremely poor sequence conservation between orthologous PMEL RPT domains, there are notable similarities. As discussed above, the repeat domain is highly O-glycosylated in vivo, and all of our clade representative sequences have threonine/serine residues evenly spaced to form a “[TS] ladder” (Figure 9, asterisks). The pharmacological inhibition of O-glycosylation using benzyl-2-acetamido-2-deoxy-α-d-galactopyranoside causes a collapsed PMEL amyloid [22], highlighting the importance of these residues. However, it is not currently known if the precise spacing of these residues is also important for their function. Furthermore, 7/10 of the RPT domains in fish lacked a clear [TS] ladder (Figure S6, Supplementary Materials). It would be interesting to determine if the PMEL amyloid structure of these species is more densely packed than those with clear [TS] ladders and whether the RPT domain of these fish can rescue the loss of the charged residues in PMEL fibril formation.

The glutamic acid residues in the *Pmel* RPT have been speculated to form hydrogen bonds similar to β-zippers of other amyloids in low-pH conditions [102]. However, the recently discovered accessory role of the PMEL RPT domain may instead implicate the regularly spaced, negatively charged residues with supporting the fibril arrangement of the amyloid. Evenly spaced glutamic acid/aspartic acid residues are a common feature of most (7/8) clade representative repeats (Figure 9), as well as most mammals (9/11) and fish (9/10) (Figures S3 and S6, Supplementary Materials). However, this pattern of [ED] ladders is rarely observed in bird (Figure S4) and reptile (Figure S5) species, even when negatively charged residues are present. More work is required to determine the functional significance of these negatively mammalian RPT domains.

### 3.8. A Strong Evolutionary Pressure for Uniform Repeat Unit Sequence, Length and E/D Ladder Conservation Is Evident in Teleost Fish

**Figure 10:**
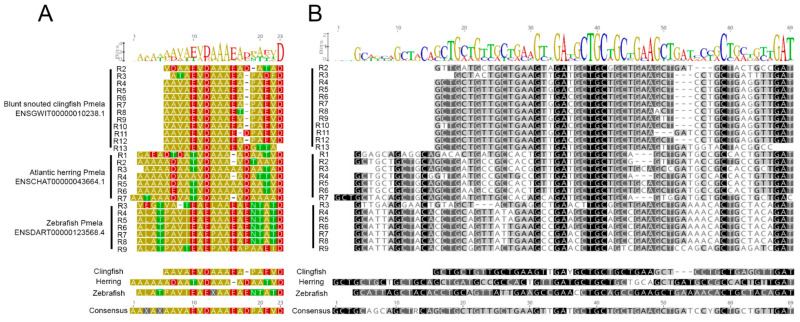
Conservation and divergence of PMEL RPT domains in fish. Amino acid (**A**) and cDNA (**B**) sequences of three teleost fish Pmela repeat domains were aligned to accentuate the insertions or deletions that differentiate the repeat unit structures of these separate species. Common sequence logos are shown above the alignments, with individual species and combined consensus sequences below. An “X” represents less than 50% consensus. (**A**) Residues are color-coded for the polarity of their amino acid side chains (yellow, nonpolar; green, uncharged polar; red, acidic; blue, basic). (**B**) The corresponding cDNA is color-coded by similarity (from darkest to lightest: 100%, >80%, >60% and <60%). An “R” represents a purine A/G, and a “Y” represents a pyrimidine C/T.

An interesting observation made during the analysis of the RPT domain sequences was that some species had near-perfect repeating protein sequences. For example, the RPT domain of zebrafish (*Danio rerio*), blunt-snouted clingfish (*Gouania willdenowi*) and Atlantic herring (*Clupea harengus)* each possess many repeat units that are almost perfectly identical in amino acid sequence within the species. However, comparing the near-perfect regions between species reveals that these closely related species underwent considerable sequence divergences (Figure 10A). We therefore examined these species in more detail at the amino acid and nucleotide levels to determine whether the evolutionary mechanisms could be deduced from the sequences. Firstly, the repeat unit numbers are different between all three species, suggesting an expansion or contraction of the tandem repeats after their divergence. Secondly, the lengths of the repeats are different between species, with a 22-mer in zebrafish, a 17-mer in clingfish and 23-mer repeat in herring. This variability cannot be explained at the DNA level by a frameshift event (since the motif V/A DAAA is common to all three species; a frameshift would shift the codon and disrupt this motif in subsequent repeats). Instead, each clingfish repeat lacks a total of 15 nucleotides per unit at the beginning of each repeat and a 3-bp deletion surrounded by a highly conserved GATGC motif upstream and GCT motif downstream that is found in 23/25 of the repeats (Figure 10B). For these nucleotides to have been deleted in clingfish (or inserted in herring and zebrafish), these mutations would have had to occur multiple times over (once per repeat unit). A more parsimonious explanation is therefore that these changes occurred after the divergence of these species from the last common ancestor but before there was expansion of the number of repeat units. Alternatively, it could be explained by very strong purifying/positive selection pressure to return to a uniform repeat unit length and consistent sequence. This area of the genome could also be particularly prone to DNA duplication, gene conversion and mobile elements of unequal recombination/crossing over events. 

One might speculate that these near-perfect repeat units confer an evolutionary advantage to the species examined or that imperfect repeats confer an evolutionary disadvantage. However, a far greater number of sequences from individual animals than are currently available would be required to test this hypothesis. Despite this divergence, these three species did have regularly spaced D/E ladders within all of the repeats, separated by speciation events. 

## 4. Conclusions

The PKAT family of genes are linked by a common ancestor and share a common domain architecture. However, the functional roles of PMEL and GPNMB have few commonalities, whereas practically nothing is known about the most ancient member, TMEM130. In this manuscript, we investigated the commonalities in the mutant phenotypes, evolutionary origin and functional domains between GPNMB and PMEL and analyzed the major difference: the RPT domain. Furthermore, we established that TMEM130 is well-suited to serve as an outgroup for future analyses comparing PMEL and GPNMB functions. We hope that this analysis assists with improving the understanding of the PKAT family and encourages further investigations into their evolution and diversification to perform such disparate functions.

## Figures and Tables

**Table 1:** List of animal *pmel* variants associated with disease with the domain designation, inheritance pattern and reported phenotype ^1^.

Domain	Animal	HGVS cDNA	HGVS Protein	Inheritance	Phenotype	Notes	Reference
SP	Cattle	NM_001080215.2: c.50_52del	NP_001073684.2: p.(Leu18del)	dominant	dilute coat	Highland/Galloway (c.64G > A) allele, interacts with MC1R (e) allele	Schmutz 2013
SP	Cattle	NM_001080215.2: c.64G > A	NP_001073684.2: p.(Gly22Arg)	dominant	dilute coat	Charolais (Dc) allele	Kühn 2007
NTR	Mouse	NM_021882.4: c.74_331del	NP_068682.2: p.(Gly25_Asn110del)	recessive	dilute coat and tail, loss of rod-shape in melanosomes (including uveal melanocytes and RPE cells)	null allele	Hellström 2011
NTR	Quail	XM_032441057.1: c.271T > G, 353G > A, 446G > A	XP_032296948.1: p.(Ser91Ala), (Arg118His), (Trp149Ter)	recessive	yellowish plumage	“L” strain	Ishishita 2018
PKD	Chicken	-	“del.280-284PTVT” relative to AY636124.1	dominant	grayish plumage	Smoky allele, modifies Dominant White mutation	Kerje 2004
RPT	Zebrafish	NM_001045330.1: c.1474G > T	NP_001038795.1: p.(Glu492Ter)	n/a	hypopigmented body and RPE, vision defects	“fading vision” (fdv) mutant	Schonthaler 2005
TM	Chicken	-	“ins.723-725WAP” relative to AY636124.1	dominant	white plumage	Dominant white allele, coincident with N399D variant upstream of RPT	Kerje 2004
TM	Chicken	-	“del.731-7135LGTAA” relative to AY636124.1	dominant	brown/khaki plumage	Dun allele, coincident with A35V, G105S, and R740C variants in NTR or Cyt domains	Kerje 2004
TM	Horse	NM_001163889.1: c.1849C > T	NP_001157361.1: p.(Arg617Cys)	dominant	silver mane and tail, body dapples, MCOA syndrome	Multiple Congenital Ocular Anomalies	Andersson 2013
Cyt	Dog	“SINE insertion in final intron”	(predicted to affect splicing)	dominant	merle (diluted) coat pattern, various auditory and ocular defects	variable oligo(dA) length affects phenotype	Clark 2006; Murphy 2018
Cyt	Mouse	NM_021882.4: c.1805insA	NP_068682.2: p.(Trp602Ter)	recessive	silver coat	reduction in melanocyte density	Martínez-Esparza 1999
Cyt	Zebrafish	NM_001045330.1: c.2426_2436del	NP_001038795.1: p.(Arg810fs)	recessive	global hypopigmentation, ocular defects	pmelaua5022 allele	Lahola-Chomiak 2018

^1^ SP = signal peptide, NTR = N-terminal region, PKD = polycystic kidney disease domain, RPT = repeat domain, TM = transmembrane domain, Cyt = cytosomal portion, RPE = retinal pigmented epithelium and MCOA = multiple congenital ocular anomalies.

**Table 2:** List of published human *PMEL* mutations with the domain designation, inheritance pattern and reported phenotype ^2^.

Domain	HGVS cDNA	HGVS Protein	Inheritance	Phenotype	Notes	Reference
NTR	NM 001200054.1 c.332A	NP 001186983.1: p.(Asn111Ser)	putative dominant	PDS	singleton	Lahola-Chomiak 2018
CAF	NM 001200054.1 c.523G > A	NP 001186983.1: p.(Gly175Ser)	dominant	PDS / PG	13 member family	Lahola-Chomiak 2018
RPT	NM 001200054.1 c.974G > T	NP 001186983.1: p.(Gly325Val)	putative dominant	PDS / PG	singleton	Lahola-Chomiak 2018
RPT	NM 001200054.1 c.994G > A	NP 001186983.1: p.(Val332Ile)	putative dominant	PDS	singleton	Lahola-Chomiak 2018
RPT	NM 001200054.1 c.1019C > T	NP 001186983.1: p.(Ala340Val)	putative dominant	PDS / PG	2 member family	Lahola-Chomiak 2018
RPT	NM 001200054.1 c.1110G > C	NP 001186983.1: p.(Glu370Asp)	putative dominant	PDS / PG	singleton x3	Lahola-Chomiak 2018
RPT	NM 001200054.1 c.1112G > C	NP 001186983.1: p.(Ser371Thr)	putative dominant	PDS	singleton	Lahola-Chomiak 2018
RPT	NM 001200054.1 c.1166T > C	NP 001186983.1: p.(Leu389Pro)	putative dominant	PDS	singleton x3	Lahola-Chomiak 2018
Cyt	NM 001200054.1 c.1921_1926del	NP 001186983.1: p.(Ser641_Ser642del)	putative dominant	PDS / PG	singleton	Lahola-Chomiak 2018

^2^ NTR = N-terminal region, CAF = core amyloid fragment, RPT = repeat domain, Cyt = cytosomal portion, PDS = pigment dispersion syndrome and PG = pigmentary glaucoma.

**Table 3:** List of the human *GPNMB* variants associated with disease with the domain designation, inheritance pattern and reported phenotype ^3^.

Domain	HGVS cDNA	HGVS Protein	Inheritance	Phenotype	Notes	Reference
NTR/CAF	NM_001005340.2: c.296del, c.565C > T	NP_001005340.1: p.(Asn99ThrfsTer2) p.(Arg189Ter)	homozygous; putative recessive	ACD	Han Chinese	Yang 2018
CAF	NM_001005340.2: c.522C > G	NP_001005340.1: p.(Ile174Met)	homozygous; recessive	ACD	consanguineous; Pakistani	Rahman 2021
CAF	NM_001005340.2: c.565C > T	NP_001005340.1: p.(Arg189Ter)	homozygous; putative recessive	ACD	Han Chinese	Yang 2018
CAF	NM_001005340.2: c.565C > T	NP_001005340.1: p.(Arg189Ter)	homozygous; recessive	ACD	Chinese	Sha 2021
CAF/CAF	NM_001005340.2: c.565C > T, c.660T > G	NP_001005340.1: p.(Arg189Ter) p.(Tyr220Ter)	compound heterozygous; recessive	ACD, skin blisters	Han Chinese	Yang 2018
CAF/btw PKD + KLD	NM_001005340.2: c.565C > T, c.1092del	NP_001005340.1: p.(Arg189Ter) p.(Pro365LeufsTer20)	compound heterozygous; semi-dominant	ACD	Taiwanese; Han Chinese	Onoufriadis 2019; Yang 2018
CAF/KLD	NM_001005340.2: c565C > T, c.1273T > C	NP_001005340.1: p.(Arg189Ter) p.(Cys425Arg)	compound heterozygous; recessive	ACD	Thai	Chiu 2021
CAF	NM_001005340.2: c.700 + 5G > T	NP_001005340.1: p.(Asp234GlyfsTer7)	homo- / heterozygous; semi-dominant	ACD	consanguineous; Kuwaiti Bedouin	Onoufriadis 2019
CAF/PKD	NM_001005340.2: c.719_720del, c.877_880del	NP_001005340.1: p.(Val240AspfsTer24) p.(Val293ProfsTer6)	compound heterozygous; putative recessive	ACD	Han Chinese	Yang 2018
btw PKD + KLD	NM_001005340.2: c.1124G > T	NP_001005340.1: p.(Gly375Val)	homozygous; recessive	ACD	consanguineous; Pakistani	Rahman 2021
KLD	NM_001005340.2 c.1274G > C	NP_001005340.1: p.(Cys425Ser)	homo- / heterozygous; semi-dominant	ACD	Filipino	Onoufriadis 2019

**^3^** NTR = N-terminal region, CAF = core amyloid fragment, PKD = polycystic kidney disease domain, KLD = Kringle-like domain, Cyt = cytosomal portion and ACD = amyloidosis cutis dyschromica.

**Table 4:** List of published animal *Gpnmb* mutations with the domain designation, inheritance pattern and reported phenotype ^4^.

Domain	Animal	HGVS cDNA	HGVS Protein	Inheritance	Phenotype	Notes	Reference
NTR	Mouse	NM_053110.4: c.653C > T	NP_444340.3: p.(Arg150Ter)	recessive	PDS, PG	DBA/2J (D2)	Anderson 2002
btw CAF + PKD	Zebrafish	ENSDART00000090883.6: c.799C > T	ENSDARP00000085316.5 P.(Gln267Ter)	N/A	none reported	ZFIN ID: ZDB-ALT-130411-2760	Dooley 2019
PKD	Zebrafish	ENSDART00000090883.6: c.854G > A	ENSDARP00000085316.5 p.(Trp285Ter)	N/A	none reported	ZFIN ID: ZDB-ALT-130411-2835	Dooley 2019

**^4^** NTR = N-terminal region, CAF = core amyloid fragment, PKD = polycystic kidney disease, PDS = pigment dispersion syndrome and PG = pigmentary glaucoma.

## Data Availability

Data is contained within the article or Supplementary Material.

## Supplementary Materials

The following are available online: Figure S1: Gene synteny analysis of the *pmel* and *gpnmb* inparalogs in lamprey. Figure S2: Clade representative alignments of the *tmem130* KLD. Figure S3: Cladogram of the mammalian RPT domains. Figure S4: Cladogram showing avian species Pmel RPT domains. Figure S5: Cladogram showing the amphibian and reptile Pmel RPT domains. Figure S6: Cladogram showing the fish Pmel RPT domain. Table S1: Percentage sequence similarity of the human PTHR11861 members at the cDNA level. Table S2: Percentage sequence similarity of the human PTHR11861 members at the protein level. Table S3: Functional domains predicted in human TMEM130 (ENST00000416379.6), GPNMB (ENST00000647578.1) and PMEL (ENST00000548493.5) by the InterPro server. Table S4: Clade-representative paralog sequences used. Table S5: Paralogous genes from humans, chickens or thorny skate that were found in neighboring lamprey gpnmb, pmel-like, pmel or gpnmb-like. Table S6: Additional PMEL paralog sequences used for the RPT domain predictions.

## Sample Availability

Zebrafish mutants are available from the authors (W.T.A.) for supply to qualified requestors.
