# Supplementary material for: Functional Domains and Evolutionary History of the PMEL and GPNMB Family Proteins
Source: Molecules. 2021 Jun 9;26(12):3529. doi: 10.3390/molecules26123529 (PMC8273697; doi:10.3390/molecules26123529)
Supplement: Supplementary file 1 [file molecules-26-03529-s001.zip › molecules-1208781.pdf]

Supplemental materials for:

Functional domains and evolutionary history of the PMEL and GPNMB family proteins

Paul W. Chrystal[1,2,*], Tim Footz[1,*], Elizabeth D. Hodges[2], Justin A. Jensen[2], Michael A. Walter[1,4], W. Ted Allison[1,2,3,4]

[1] Department of Medical Genetics, University of Alberta, Edmonton AB, T6G 2R3, CANADA;
[2] Department of Biological Sciences, University of Alberta, Edmonton AB, T7Y 1C4, CANADA;
[3] Centre for Prions & Protein Folding Disease, University of Alberta, Edmonton AB, T6G 2M8, CANADA
[4] Correspondence to WTA (ted.allison@ualberta.ca); MAW (mwalter@ualberta.ca)
* Co-first authors.

Consisting of:

- Six Supplemental Tables, and
- Six Supplemental Figures

Table S1. Percentage sequence identity of human PTHR11861 members at the cDNA level.

**PKAT *family cDNA alignment***			
	**TMEM130**	**GPNMB**	**PMEL**
**TMEM130**	100	-	-
**GPNMB**	32.476	100	-
**PMEL**	38.713	33.202	100

Table S2. Percentage sequence identity of human PTHR11861 members at the protein level.

**PKAT *family protein alignment***			
	**TMEM130**	**GPNMB**	**PMEL**
**TMEM130**	100	-	-
**GPNMB**	17.327	100	-
**PMEL**	13.663	24.929	100

Table S3. Functional domains predicted in human TMEM130 (ENST00000416379.6), GPNMB (ENST00000647578.1) and PMEL (ENST00000548493.5) by InterPro server.

Human TMEM130 | **TMEM130-203** | **ENST00000416379.6**

Descriptor	Start	End	Length	Source	Ref no.
Signal Peptide	1	27	27	PHOBIUS entry	SIGNAL_PEPTIDE (1)
Signal peptide N-region	1	11	11	PHOBIUS entry	SIGNAL_PEPTIDE_N_REGION (6)
SignalP-noTM	1	28	28	SIGNALP_EUK entry	SignalP-noTM (8)
Signal peptide H-region	12	22	11	PHOBIUS entry	SIGNAL_PEPTIDE_H_REGION (2)
Tmhelix	12	31	20	TMHMM entry	TMhelix (7)
Signal peptide C-region	23	27	5	PHOBIUS entry	SIGNAL_PEPTIDE_C_REGION(4)
PKD	144	210	67	CDD entry	cd00146
Ig-like_fold	157	225	69	InterPro homologous superfamily	IPR013783
Immunoglobulins	157	225	69	CATH-Gene3D	G3DSA:2.60.40.10
PKD domain	171	213	43	SUPERFAMILY	SSF49299
PKD_dom_sf	171	334	164	InterPro homologous superfamily	IPR035986
PKD_dom	177	210	34	InterPro domain	IPR000601
PKD_dom	177	210	34	PROSITE profiles	PS50093
PKD domain	293	334	42	SUPERFAMILY	SSF49299
Transmembrane region	340	362	23	PHOBIUS entry	TRANSMEMBRANE (5)
Tmhelix	340	362	23	TMHMM entry	Tmhelix (9)
Cytoplasmic domain	363	435	73	PHOBIUS entry	CYTOPLASMIC_DOMAIN (3)
Non cytoplasmic domain	28	339	312	PHOBIUS entry	NON_CYTOPLASMC_DOMAIN (10)
MELANOCYTE PROTEIN PMEL 17-related	23	435	413	PANTHER entry	PTHR11861
TRANSMEMBRANE PROTEIN 130	23	435	413	PANTHER entry	PTHR11861:SF10

Human GPNMB | **GPNMB-215** | **ENST00000647578.1**

Descriptor	Start	End	Length	Source	Ref no.
Signal peptide N-region	1	2	2	PHOBIUS entry	SIGNAL_PEPTIDE_N_REGION (2)
Signal Peptide	1	21	21	PHOBIUS entry	SIGNAL_PEPTIDE (6)
SignalP-noTM	1	22	22	SIGNALP_EUK entry	SignalP-noTM (4)
Signal peptide H-region	3	14	12	PHOBIUS entry	SIGNAL_PEPTIDE_H_REGION (5)
Signal peptide C-region	15	21	7	PHOBIUS entry	SIGNAL_PEPTIDE_C_REGION (9)
PKD/Chitinase_dom	250	402	153	InterPro domain	IPR022409
PKD_9	250	402	153	SMART	SM00089
PKD	256	319	64	CDD entry	cd00146
Ig-like_fold	257	321	65	InterPro homologous superfamily	IPR013783
Immunoglobulins	257	321	65	CATH-Gene3D	G3DSA:2.60.40.10
PKD_dom_sf	271	316	46	InterPro homologous superfamily	IPR035986
PKD domain	271	316	46	SUPERFAMILY	SSF49299

PKD_dom	277	319	43	InterPro domain	IPR000601
PKD	277	315	39	PROSITE profiles	PS50093
PKD	279	319	41	Pfam	PF00801
Tmhelix	513	535	23	TMHMM entry	Tmhelix (8)
Transmembrane region	516	535	20	PHOBIUS entry	TRANSMEMBRANE (7)
Cytoplasmic domain	536	588	53	PHOBIUS entry	CYTOPLASMIC_DOMAIN (3)
Non cytoplasmic domain	22	515	494	PHOBIUS entry	NON_CYTOPLASMIC_DOMAIN (1)
TRANSMEMBRANE GLYCOPROTEIN NMB	1	583	583	PANTHER entry	PTHR11861:SF11
MELANOCYTE PROTEIN PMEL 17-RELATED	1	583	583	PANTHER entry	PTHR11861

Human PMEL				PMEL-204	ENST00000548493.5
**Descriptor**	**Start**	**End**	**Length**	**Source**	**Ref no.**
Signal peptide N-region	1	7	7	PHOBIUS entry	SIGNAL_PEPTIDE_N_REGION
SignalP-TM	1	22	22	SIGNALP_GRAM_POSITIVE entry	SignalP-TM (10)
Signal Peptide	1	23	23	PHOBIUS entry	SIGNAL_PEPTIDE (8)
SIGNALP_EUK entry	1	24	24	SignalP_EUK entry	SignalP-noTM (4)
Signal peptide H-region	8	19	12	PHOBIUS entry	SIGNAL_PEPTIDE_H
Signal peptide C-region	20	23	4	PHOBIUS entry	SIGNAL_PEPTIDE_C_REGION (9)
Non cytoplasmic domain	24	595	572	PHOBIUS entry	NON_CYTOPLASM
Ig-like_fold	226	304	79	InterPro homologous superfamily	IPR013783
Immunoglobulins	226	304	79	CATH-Gene3D	G3DSA:2.60.40.10
PKD/Chitinase_dom	229	311	83	Interpro Domain	IPR022409
PKD_9	229	311	83	SMART	SM00089
PKD	233	295	63	CDD entry	cd00146
PKD_Dom	233	300	68	Interpro Domain	IPR000601
PKD	233	300	68	Pfam	PF00801
PKD_dom_sf	252	289	38	InterPro homologous superfamily	IPR035986
PKD domain	252	289	38	SUPERFAMILY	SSF49299
PKD	255	292	38	PROSITE profiles	PS50093
disorder_prediction	302	353	52	MOBIDB_LITE entry	mobidb-lite (3)
Tmhelix	593	615	23	TMHMM entry	TMhelix (7)
Transmembrane region	596	616	21	PHOBIUS entry	TRANSMEMBRANE
Cytoplasmic domain	617	661	45	PHOBIUS entry	CYTOPLASMIC_DOMAIN (11)
Melanocyte protein PMEL 17 -related	4	661	658	PANTHER entry	PTHR11861
Melanocyte protein PMEL	4	661	658	PANTHER entry	PTHR11861:SF1

Table S4. Clade-representative paralog sequences used.

	tmem130	gpnmb	pmel
**Human (Homo sapiens)**	ENST00000416379.6	NP_001005340.1	ENST00000548493.5
**Chicken (Gallus gallus)**	ENSGALT00000041067.4	ENSGALT00000017821.6	ENSGALG00000035350
**Common lizard (Zootoca vivipara)**	XP_034987520.1	XP_034985369.1	XP_034959827.1
**Tropical clawed frog (Xenopus tropicalis)**	XP_012826896.2	NP_001124514.1	XP_002934561.3
**Coelacanth (Latimeria chalumnae)**	XP_006000848.2	XP_005997158.1	XP_005986276.1
**Zebrafish (Danio rerio)**	ENSDART00000169019.3	ENSDART00000090883.6	ENSDART00000123568.4
			ENSDART00000046268.7
**Thorny skate (Amblyraja radiata)**	XP_032896444.1	XP_032902250.1	XP_032871492.1
**Sea lamprey (Petromyzon marinus)**		XP_032818521.1	XP_032813209.1
**Sea lamprey (Petromyzon marinus)**		XP_032807179.1	XP_032831262.1
**European starfish (Asterias rubens)**	XP_033631068.1		
**Acorn worm (Saccoglossus kowalevskii)**	XP_002739013.1		
**Common Spider (Parasteatoda tepidariorum)**	XP_015915338.1		
**Cauliflower coral (Pocillopora damicornis)**	RMX49586.1		
**Trichoplax (Trichoplax adhaerens)**	RDD42042.1		

Table S5. Paralogous genes from human, chicken or thorny skate that were found neighbouring lamprey gpnmb, pmel-like, pmel or gpnmb-like.

H. sapiens Ch 7 [GPNMB]		H. sapiens Ch 12 [PMEL]		G. gallus Ch 2 [gpnmb]		G. gallus Ch 33 [pmel]		A. radiata Ch2 [gpnmb]		A. radiata Ch 46 [pmel]	
**Gene symbol**	**Accession**	**Gene symbol**	**Accession**	**Gene symbol**	**Accession**	**Gene symbol**	**Accession**	**Gene symbol**	**Accession**	**Gene symbol**	**Accession**
RPA3	ENSG00000106399	RACGAP1P	ENSG00000257331	CHPF2	ENSGALG00000036477	TMBIM6	ENSGALG00000031426	lec1	NC_045957.1	d63	NC_046001.1
ICA1	ENSG00000003147	SLC48A1	ENSG00000211584	ASB10	ENSGALG00000032845	TFCP2	ENSGALG00000035530	ars1	NC_045957.1	fcp2	NC_046001.1
AGMO	ENSG00000187546	ASB8	ENSG00000177981	FASTK	ENSGALG0000003058	SLC4A8	ENSGALG00000031274	tgb1	NC_045957.1	4galnt1	NC_046001.1
SOSTDC1	ENSG00000171243	LMBR1L	ENSG00000139636	SLC4A2	ENSGALG00000034284	FAIM2	ENSGALG00000031615	ard3	NC_045957.1	acgap1	NC_046001.1
ANKMY2	ENSG00000106524	TUBA1A	ENSG00000167552	IBA57	ENSGALG00000005328	SPATS2	ENSGALG00000033957	itrm1	NC_045957.1	ym1	NC_046001.1
BZW2	ENSG00000136261	SPATS2	ENSG00000123352	ARF1	ENSGALG00000005393	MCRS1	ENSGALG00000035203	dhaf3	NC_045957.1	mbr1l	NC_046001.1
AGR2	ENSG00000106541	MCRS1	ENSG00000187778	CCDC12	ENSGALG00000005487	CD63	ENSGALG00000055094	pp1r9a	NC_045957.1	a2g4	NC_046001.1
TMEM196	ENSG00000173452	TMBIM6	ENSG00000139644	SETD2	ENSGALG00000042051	GDF11	ENSGALG00000036806	et1	NC_045957.1	dk2	NC_046001.1
NUP42	ENSG00000136243	TFCP2	ENSG00000135457	KLHL18	ENSGALG00000005582	SARNP	ENSGALG00000030342	lp2	NC_045957.1	mel	NC_046001.1
GPNMB	ENSG00000136235	SLC4A8	ENSG00000050438	DLEC1	ENSGALG00000005826	RACGAP1	ENSGALG00000033231	abbr2	NC_045957.1	crs1	NC_046001.1
MALSU1	ENSG00000156928	MFSD5	ENSG00000182544	U6	ENSGALG00000025663	SLC48A1	ENSGALG00000036380	fx1	NC_045957.1	pats2	NC_046001.1
IGF2BP3	ENSG00000136231	AAAS	ENSG00000094914	CRYGN	ENSGALG00000006189	ATP5F1B	ENSGALG00000038801	hmp5	NC_045957.1	lc4a8	NC_046001.1
CCDC126	ENSG00000169193	ATF7	ENSG00000170653	RHEB	ENSGALG00000039880	TIMELESS	ENSGALG00000034688	ag1	NC_045957.1	gap2	NC_046001.1
NPY	ENSG00000122585	COPZ1	ENSG00000111481	GALNT11	ENSGALG00000006233	CS	ENSGALG00000030466	trr	NC_045957.1	arnp	NC_046001.1
SNX10	ENSG00000086300	CD63	ENSG00000135404	NOM1	ENSGALG00000006437	CDK2	ENSGALG00000032699	rp44	NC_045957.1	df11	NC_046001.1
HOXA1	ENSG00000105991	GDF11	ENSG00000135414	LARP4B	ENSGALG00000006672	PMEL	ENSGALG00000035350	tx17	NC_045957.1	najc14	NC_046001.1
HOXA4	ENSG00000197576	SARNP	ENSG00000205323	PITRM1	ENSGALG00000007036	PYM1	ENSGALG00000046169	ec61b	NC_045957.1	imeless	NC_046001.1
HOXA9	ENSG00000078399	DNAJC14	ENSG00000135392	PARD3	ENSGALG00000007125	TUBA1A	ENSGALG00000037953	ba57	NC_045957.1	rnas-cga	NC_046001.1
HOXA10	ENSG00000253293	PYM1	ENSG00000170473	ITGB1	ENSGALG00000007145	LMBR1L	ENSGALG00000037665	rygn	NC_045957.1		
HOXA11	ENSG00000005073	PMEL	ENSG00000185664	SVIL	ENSGALG00000007331	MFSD5	ENSGALG00000033927	heb	NC_045957.1		
JAZF1	ENSG00000153814	CDK2	ENSG00000123374	ARMC4	ENSGALG00000007417	ASB8	ENSGALG00000037396	alnt11	NC_045957.1		
FKBP14	ENSG00000106080	PA2G4	ENSG00000170515	RAB18	ENSGALG00000007435	AAAS	ENSGALG00000032843	om1	NC_045957.1		

GARS1	ENSG00000106105	CS	ENSG00000062485	YME1L1	ENSGALG00000007492	COPZ1	ENSGALG00000043336	arp4b	NC_045957.1		
LSM5	ENSG00000106355	CNPY2	ENSG00000257727	MASTL	ENSGALG00000007507			di1	NC_045957.1		
AVL9	ENSG00000105778	TIMELESS	ENSG00000111602	ACBD5	ENSGALG00000007519			vil	NC_045957.1		
BBS9	ENSG00000122507	ATP5F1B	ENSG00000110955	ABI1	ENSGALG00000007547			zw2	NC_045957.1		
BMPER	ENSG00000164619	NDUFA4L2	ENSG00000185633	PDSS1	ENSGALG00000007559			nkmy2	NC_045957.1		
VPS41	ENSG00000006715	MARS1	ENSG00000166986	ABCB1	ENSGALG00000008912			ostdc1	NC_045957.1		
RALA	ENSG00000006451	B4GALNT1	ENSG00000135454	SRI	ENSGALG00000008985			rppa	NC_045957.1		
SUGCT	ENSG00000175600	AGAP2	ENSG00000135439	AKAP9	ENSGALG00000040724			gmo	NC_045957.1		
INHBA	ENSG00000122641	OTOGL	ENSG00000165899	KRIT1	ENSGALG00000042651			ca1	NC_045957.1		
PSMA2	ENSG00000106588	APAF1	ENSG00000120868	PEX1	ENSGALG00000009410			pa3	NC_045957.1		
MRPL32	ENSG00000106591			BET1	ENSGALG00000024485			mem196	NC_045957.1		
ADCY1	ENSG00000164742			PPP1R9A	ENSGALG00000009686			rnad-guc	NC_045957.1		
UPP1	ENSG00000183696			RPA3	ENSGALG00000010700			up42	NC_045957.1		
LANCL2	ENSG00000132434			ICA1	ENSGALG00000010708			pnmb	NC_045957.1		
VOPP1	ENSG00000154978			AGMO	ENSGALG00000010792			alsu1	NC_045957.1		
ABCB1	ENSG00000085563			CRPPA	ENSGALG00000010795			gf2bp3	NC_045957.1		
SRI	ENSG00000075142			SOSTDC1	ENSGALG00000036836			cdc126	NC_045957.1		
AKAP9	ENSG00000127914			ANKMY2	ENSGALG00000010804			py	NC_045957.1		
KRIT1	ENSG00000001631			BZW2	ENSGALG00000010809			ex1	NC_045957.1		
PEX1	ENSG00000127980			AGR2	ENSGALG00000010825			hrb	NC_045957.1		
BET1	ENSG00000105829			TMEM196	ENSGALG00000010865			arb	NC_045957.1		
PPP1R9A	ENSG00000158528			GPNMB	ENSGALG00000010949			gly1	NC_045957.1		
SDHAF3	ENSG00000196636			MALSU1	ENSGALG00000010954			ek10	NC_045957.1		
MUC17	ENSG00000169876			IGF2BP3	ENSGALG00000010961			zi2	NC_045957.1		
TPK1	ENSG00000196511			CCDC126	ENSGALG00000010976			kbp14	NC_045957.1		
SLC4A2	ENSG00000164889			NPY	ENSGALG00000010983			azf1	NC_045957.1		

FASTK	ENSG00000 164896			SNX10	ENSGALG0 0000011046			oxa11	NC_045957. 1		
ASB10	ENSG00000 146926			HOXA1	ENSGALG0 0000028095			oxa10	NC_045957. 1		
CHPF2	ENSG00000 033100			HOXA4	ENSGALG0 0000022622			oxa9	NC_045957. 1		
CRYGN	ENSG00000 127377			HOXA9	ENSGALG0 0000028983			oxa4	NC_045957. 1		
RHEB	ENSG00000 106615			HOXA10	ENSGALG0 0000026631			oxa1	NC_045957. 1		
GALNT11	ENSG00000 178234			HOXA11	ENSGALG0 0000040021			nx10	NC_045957. 1		
NOM1	ENSG00000 146909			JAZF1	ENSGALG0 0000030455			nkh	NC_045957. 1		
				FKBP14	ENSGALG0 0000011181			yco1	NC_045957. 1		
				HACL1	ENSGALG0 0000011211			lipr2	NC_045957. 1		
				BTD	ENSGALG0 0000011216			mem245	NC_045957. 1		
				ANKRD28	ENSGALG0 0000011226			rrs1l	NC_045957. 1		
				GALNT15	ENSGALG0 0000011235			dcy1	NC_045957. 1		
				OXNAD1	ENSGALG0 0000011239			pk1	NC_045957. 1		
**Syntanous w/ gpnmb**				TBC1D5	ENSGALG0 0000011251			ancl2	NC_045957. 1		
**Syntanous w/ pmel**				KCNH8	ENSGALG0 0000011262			opp1	NC_045957. 1		
				KAT2B	ENSGALG0 0000011278			acl1	NC_045957. 1		
				SGO1	ENSGALG0 0000011281			td	NC_045957. 1		
				THRB	ENSGALG0 0000011294			nkrd28	NC_045957. 1		
				RARB	ENSGALG0 0000011298			alnt15	NC_045957. 1		
				NGLY1	ENSGALG0 0000011304			xnad1	NC_045957. 1		
				NEK10	ENSGALG0 0000011322			bc1d5	NC_045957. 1		
				AZI2	ENSGALG0 0000011428			cnh8	NC_045957. 1		
				DNAJC13	ENSGALG0 0000035068			at2b	NC_045957. 1		
				ACKR4	ENSGALG0 0000041491			go1	NC_045957. 1		
				TMEM108	ENSGALG0 0000036412			bitram	NC_045957. 1		
				FYCO1	ENSGALG0 0000040212			rit1	NC_045957. 1		

				LZTFL1	ENSGALG00000054523			kap9	NC_045957.1		
				CLASP2	ENSGALG00000032647			ri	NC_045957.1		
				ARPP21	ENSGALG00000043240			ckr4	NC_045957.1		
				BMPER	ENSGALG00000029811			najc13	NC_045957.1		
				BBS9	ENSGALG00000031650			ztfl1	NC_045957.1		
				AVL9	ENSGALG00000041568			yo10	NC_045957.1		
				LSM5	ENSGALG00000041625			nf622	NC_045957.1		
				VPS41	ENSGALG00000031635			mem108	NC_045957.1		
				RALA	ENSGALG00000031893			bxl7	NC_045957.1		
				SUGCT	ENSGALG00000031758			rnag-gcc	NC_045957.1		
				INHBA	ENSGALG00000034616			dss1	NC_045957.1		
				PSMA2	ENSGALG00000039387			bi1	NC_045957.1		
				MRPL32	ENSGALG00000037258			cbd5	NC_045957.1		
				VOPP1	ENSGALG00000042773			astl	NC_045957.1		
				LANCL2	ENSGALG00000032570			me1l1	NC_045957.1		
				TPK1	ENSGALG00000040298			rmc4	NC_045957.1		
				ADCY1	ENSGALG00000031170			sma2	NC_045957.1		
				NFX1	ENSGALG00000043724			rpl32	NC_045957.1		
				RECK	ENSGALG00000036829						
				ZNF622	ENSGALG00000037171						
				FBXL7	ENSGALG00000031947						
				ANKH	ENSGALG00000012964						
				MTRR	ENSGALG00000039424						
				UPP1	ENSGALG00000013073						
				TMEM245	ENSGALG00000013138						
				FRRS1L	ENSGALG00000025792						

				BAG1	ENSGALG00000013157						
				CHMP5	ENSGALG00000013160						
				GABBR2	ENSGALG00000013200						
				ELP2	ENSGALG00000039034						
				ERP44	ENSGALG00000013452						
				STX17	ENSGALG00000013462						
				SEC61B	ENSGALG00000013569						
				GLIPR2	ENSGALG00000028821						
				MOS	ENSGALG00000042082						

Table S6. Additional PMEL paralog sequences used for RPT domain predictions.

Supplemental Figures	
**Agassiz's desert tortoise (Gopherus agassizii)**	ENSGAGT00000005719.1
**Atlantic herring (Clupea harengus)**	ENSCHAT00000043664.1
**Barn Owl (Tyto alba)**	AUD07748.1
**Barred-Tailed Pigeon (Patagioenas fasciata monilis)**	OPJ72592.1
**Blind Cavefish (Astyanax fasciatus mexicanus)**	A0A4W6F2T7
**Blunt snouted clingfish (Gouania willdenowi)**	ENSGWIT00000010238.1
**Brown Trout (Salmo trutta)**	ENSSTUT00000048284.1
**Central Bearded Dragon (Pogona vitticeps)**	XP_020638533.1
**Cod (Gadus morhua)**	ENSGMOT00000001759.1
**Cow (Bos taurus)**	NP_001073684.2
**Dog (Canis lupus familiaris)**	NP_001096686.1
**Electric Eel (Electrophorus electricus)**	XP_026888855.1
**European Rabbit (Oryctolagus cuniculus)**	CCA62427.1
**Gaboon caecilian (Geotrypetes seraphini)**	XP_033793418.1
**Great blue-spotted mudskipper (Boleophthalmus pectinirostris)**	XP_020792628.1
**Greater Horseshoe Bat (Rhinolophus ferrumequinum)**	XP_032972790.1
**Horse (Equus caballus)**	NP_001157361.1
**Japanese quail (Coturnix japonica)**	XP_032296948.1
**Lesser hedgehog tenrec (Echinops telfairi)**	XP_004700634.2
**Mouse (Mus musculus)**	NP_068682.2
**Okarito brown kiwi (Apteryx rowi)**	XP_025911704.1
**Owl Parrot (Strigops habroptila)**	XP_030366358.1
**Pachon Cavefish (Astyanax Mexicanus Pachon)**	ENSAMXT00005031725.1
**Platypus (Ornithorhynchus anatinus)**	XP_028928770.1
**Sperm Whale (Physeter catodon)**	XP_007129391.2
Tasmanian devil (Sarcophilus harrisii)	XP_031797073.1
**Tongue Sole (Cynoglossus semilaevis)**	XP_024915820.1
**Tufted Duck (Aythya fuligula)**	XP_032060670.1
**Two-lined caecilian (Rhinatrema bivittatum)**	XP_029450632.1

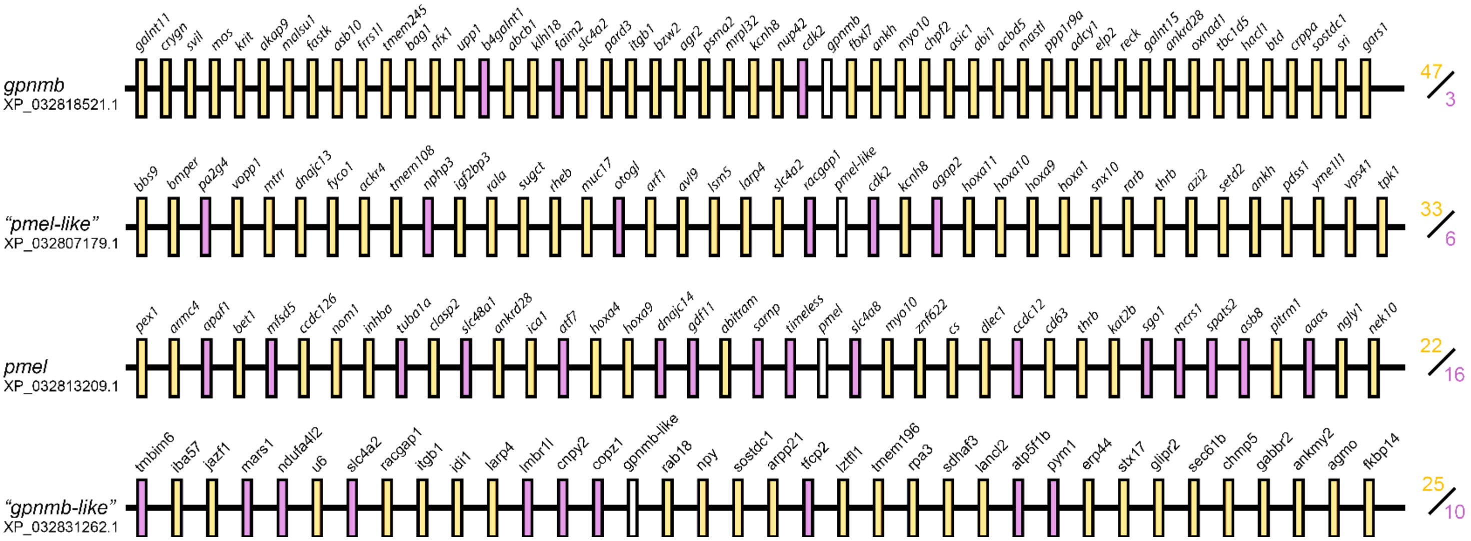

Figure S1. Gene synteny analysis of *pmel* and *gpnmb* inparalogs in lamprey. Schematic representation of lamprey genes neighbouring pmel, pmel-like, gpnmb, and gpnmb-link that have a paralogous gene in human, chicken or thorny skate. Genes that are paralogous to GPNMB in human, chicken and thorny skate are in yellow. Genes that are paralogous to PMEL in human, chicken and thorny skate are in purple.

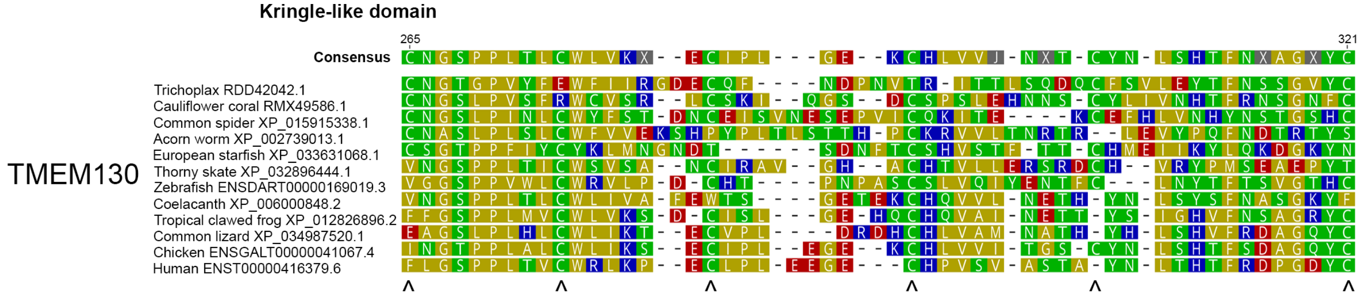

Figure S2. Clade representative alignments of the *tmem130* KLD. Sequence alignment of clade representative *TMEM130* proteins demonstrating the conservation, but not perfect identity, of the 6 cysteine residues (caret).

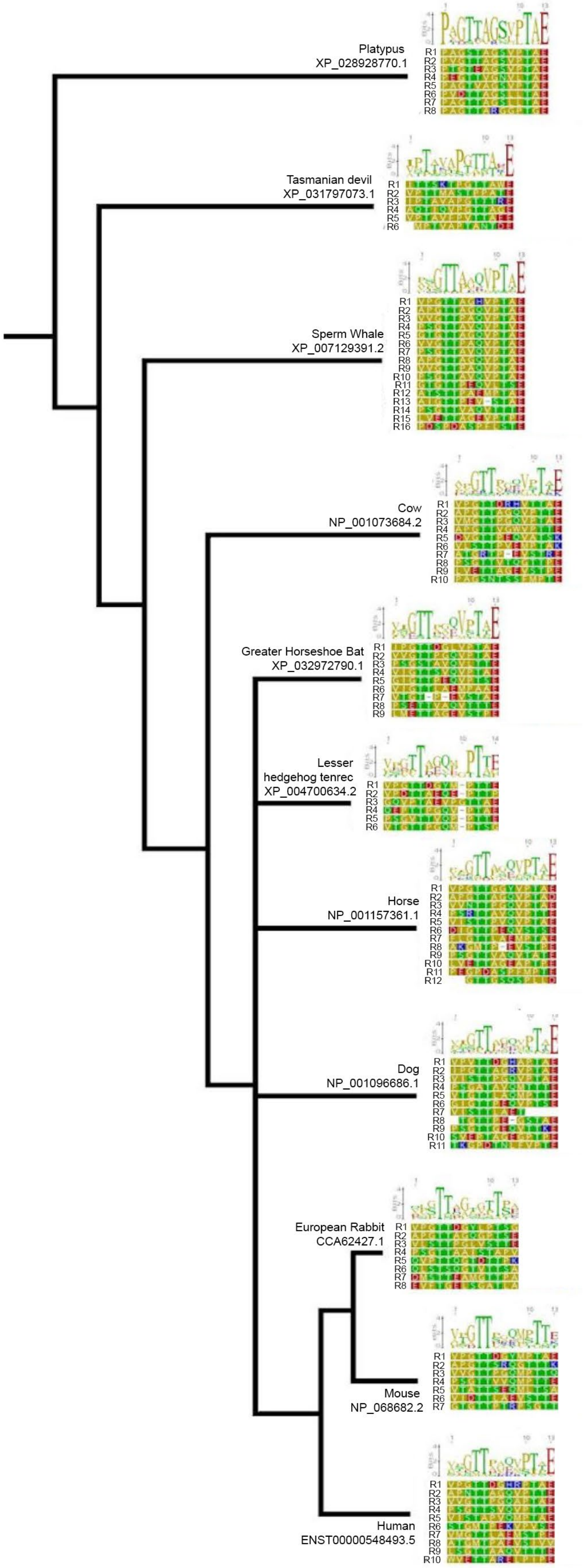

Figure S3. Cladogram of mammalian RPT domains**.** Mammalian species were selected from the following orders Monotremata, Afrosoricida, Dasyuromorphia, Cetacea, Lagomorpha, Diprotodontia, Carnivora, Artiodactyla, Rodentia, and Primates. Amino acids are color-coded according to polarity (yellow, non-polar; green, uncharged polar; red, acidic; blue, basic).

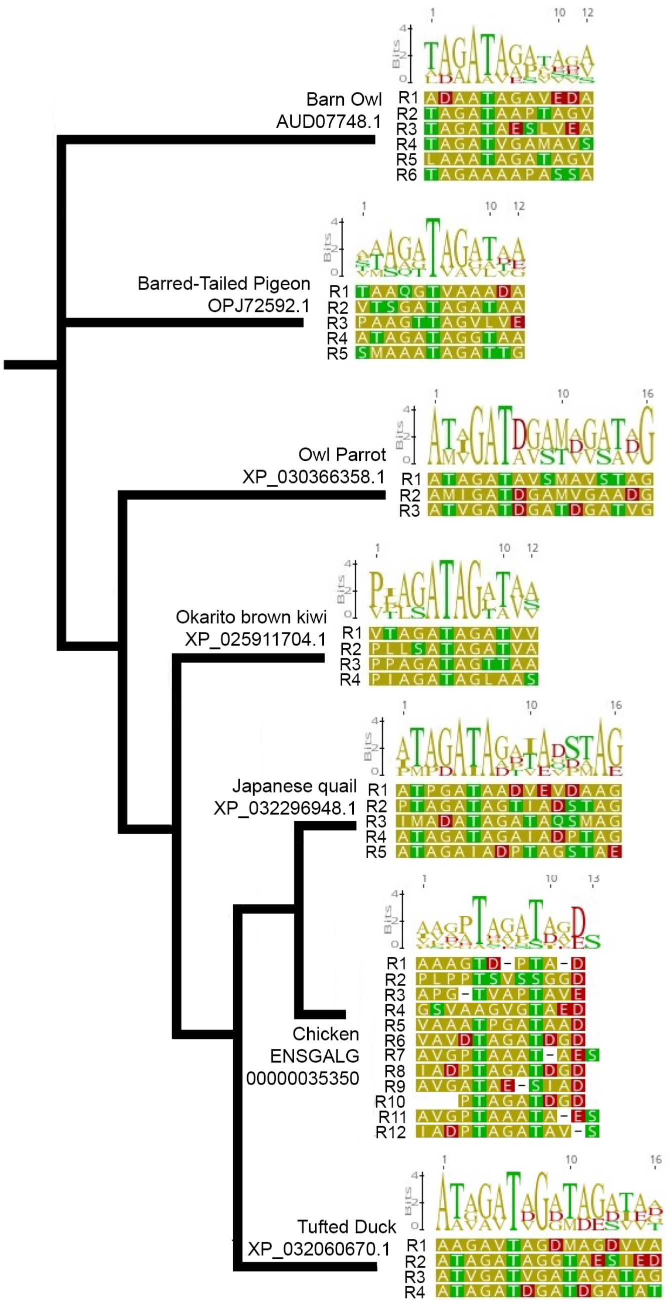

Figure S4. Cladogram **s**howing Avian species Pmel RPT domains**.** Avian species were selected from the following orders: Struthioniformes, Galliformes, Anseriformes, Psittaciformes, Strigiformes, and Columbiformes. Amino acids are color-coded according to polarity (yellow, non-polar; green, uncharged polar; red, acidic; blue, basic).

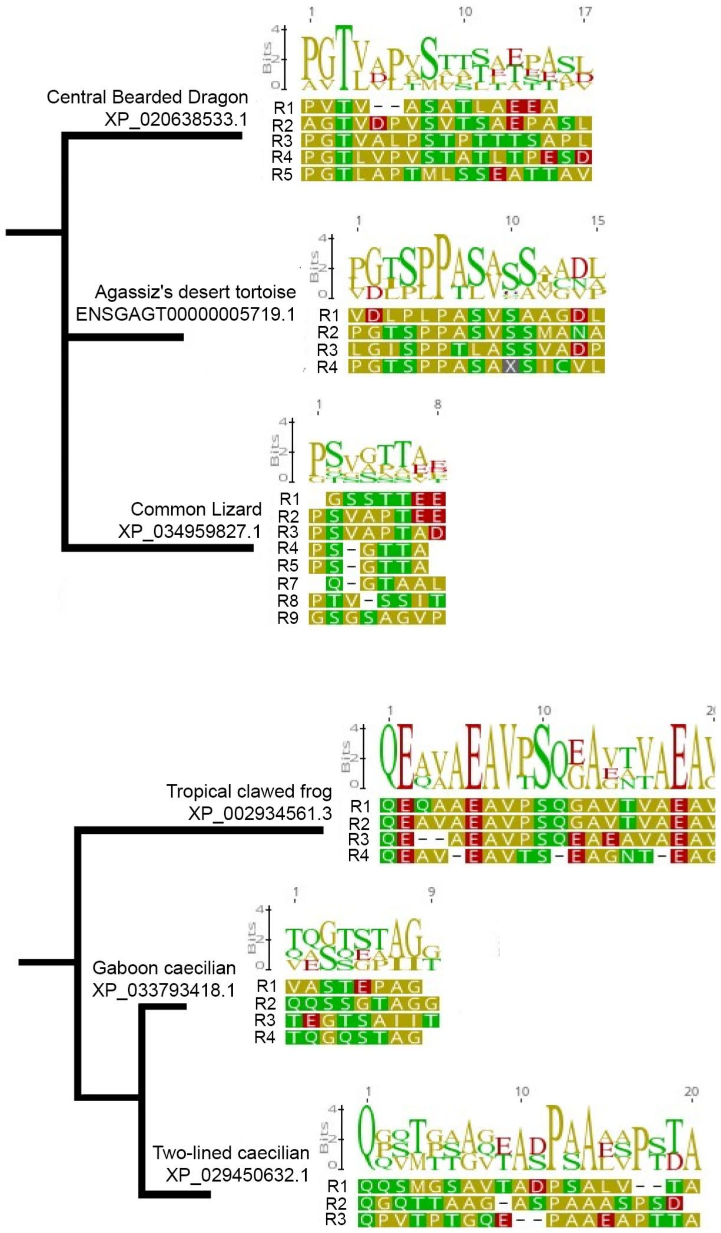

Figure S5. Cladogram showing the Amphibians and Reptiles Pmel RPT domains. A) Reptiles were selected from the following orders Testudines, Squamata. B) Amphibians were selected from the orders Anura and Gymnophiona. Amino acids are color-coded according to polarity (yellow, non-polar; green, uncharged polar; red, acidic; blue, basic).

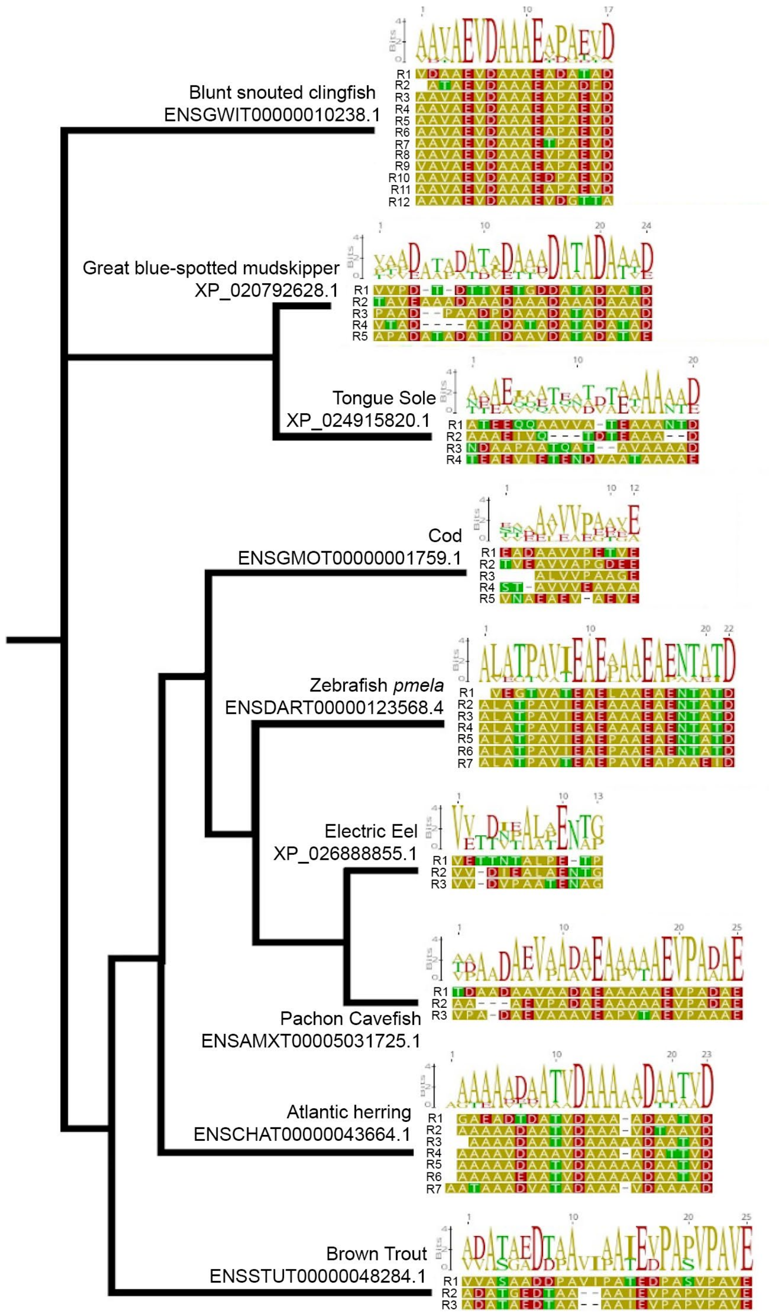

Figure S6. Cladogram showing the fishes Pmel RPT domain. Within the Teleostei class species were selected from the following orders Salmoniformes, Clupeiformes, Characiformes, Gadiformes, Pleuronectiformes, Perciformes, Gymnotiformes, Cypriniformes. Amino acids are color-coded according to polarity (yellow, non-polar; green, uncharged polar; red, acidic; blue, basic).
